# Nanotechnology Potential in Seed Priming for Sustainable Agriculture

**DOI:** 10.3390/nano11020267

**Published:** 2021-01-20

**Authors:** Anderson do Espirito Santo Pereira, Halley Caixeta Oliveira, Leonardo Fernandes Fraceto, Catherine Santaella

**Affiliations:** 1 CEA, CNRS, BIAM, Laboratory for Microbial Ecology of the Rhizosphere (LEMIRE), Aix Marseille University, UMR7265 BIAM, F-13108 Saint Paul-Lez-Durance, France; espdna@gmail.com; 2 Department of Environmental Engineering, Institute of Science and Technology of Sorocaba, São Paulo State University (UNESP), Sorocaba, SP CEP 18087-180, Brazil; 3 Department of Animal and Plant Biology, Londrina State University (UEL), Londrina, PR CEP 86047-970, Brazil; halleycaixeta@gmail.com

**Keywords:** seed nano-priming, seed coating, germination, plant resistance, nanoparticles, sustainability

## Abstract

Our agriculture is threatened by climate change and the depletion of resources and biodiversity. A new agriculture revolution is needed in order to increase the production of crops and ensure the quality and safety of food, in a sustainable way. Nanotechnology can contribute to the sustainability of agriculture. Seed nano-priming is an efficient process that can change seed metabolism and signaling pathways, affecting not only germination and seedling establishment but also the entire plant lifecycle. Studies have shown various benefits of using seed nano-priming, such as improved plant growth and development, increased productivity, and a better nutritional quality of food. Nano-priming modulates biochemical pathways and the balance between reactive oxygen species and plant growth hormones, resulting in the promotion of stress and diseases resistance outcoming in the reduction of pesticides and fertilizers. The present review provides an overview of advances in the field, showing the challenges and possibilities concerning the use of nanotechnology in seed nano-priming, as a contribution to sustainable agricultural practices.

## 1. Introduction

Agriculture is currently facing many challenges, including production losses due to pests, the effects of global climate change, and the depletion of natural resources [1,2,3]. An additional difficulty is that traditional agricultural practices have been relying on continuous application of pesticides and fertilizers, resulting in environmental contamination [3,4].

The world population is expected to increase to 9–10 billion by 2050, implying that food production will need to rise by 25–70% compared to current levels [5]. Therefore, new technologies need to be deployed in agriculture to ensure sustainability and increase productivity [2,3,6,7].

Nanotechnology has the potential to contribute to a new technology-based agricultural revolution [3,8,9]. Many nanomaterials have been developed for agricultural applications, including new solutions for soil and water remediation, as well as nanofertilizers and nanopesticides, designed to reduce the applied amounts of fertilizers and pesticides, while increasing food production and quality [2,4,8,10]. Besides, the use of nanoenabled products for crop protection can reduce significantly the impact caused by the agriculture on the environment, in this way being an ecofriendly alternative [1].

Nanomaterials, especially nanoparticles, have a range of applications for crop protection [2,3,6]. This is a major field of research that has attracted the interest of companies in the agricultural sector, resulting in the inclusion of nanoparticles in formulations [11]. The use of nanopesticides and nanofertilizers can contribute to pest control, plant nutrition, and environmentally-friendly production methods [1,3,6,12].

Recent studies have shown that nanoparticles have effects on seeds and plants [3,12,13,14]. Some nanoparticles have side effects, including the inhibition of germination or phytotoxicity in seedlings [15,16,17]. However, others can act as stimulants, improving seed metabolism, seedling vigor, and plant growth by acting in cellular signaling pathways [12,14,18]. These effects depend on nanoparticle physical-chemical properties, such as size, zeta potential, and concentration, which are factors that determine the biological responses [13,19].

These characteristics have key roles in nanoparticle uptake and translocation in plants. As example, small-sized nanoparticles cross biological barriers more efficiently [20,21,22,23]. The surface charge of the nanoparticles is also decisive. Positively or negatively charged nano-particles can be taken up by the leaves and translocated to the roots. However, only negatively charged nanos are taken up by the roots. Positive charges induce the production of mucilage, which prevents their uptake by plants [24,25,26].

**Figure 1:**
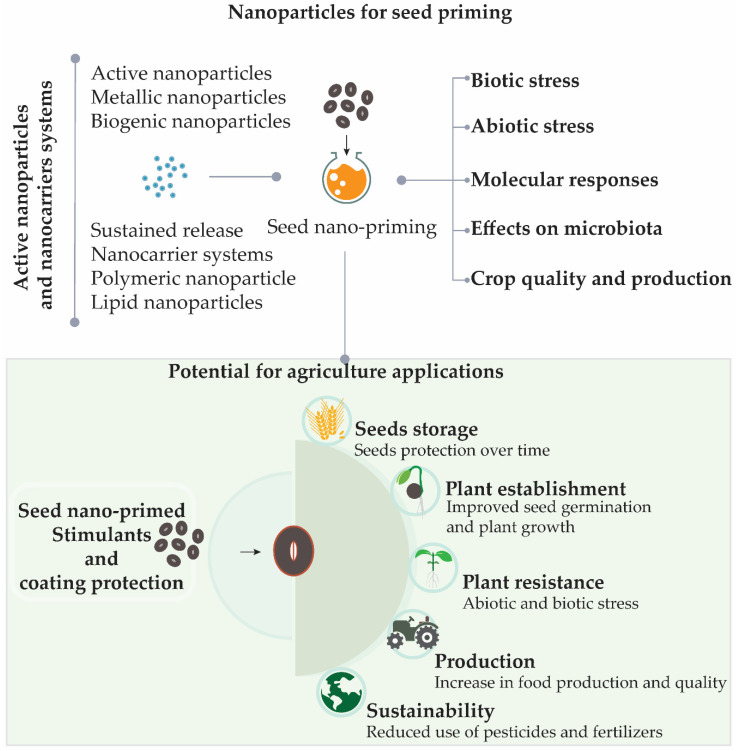
Seed nano-priming topics covered in this review and its potential benefits for sustainable agriculture. In addition to providing protection for seeds during storage, the use of seed nano-priming can result in improved establishment of plants in the soil with a reduced need for fertilizers. By growing faster, plants have an increased ability to compete with weeds for resources, consequently increasing productivity and food quality. The plants may also become more resistant to abiotic and biotic stresses, resulting in reduced use of pesticides.

Nano-priming can be applied to seeds in order to provide protection for seeds during storage, improve germination, germination synchronization, and plant growth, as well as to increase the resistance of crops to abiotic or biotic stress conditions, which can help to reduce the required quantities of pesticides and fertilizers [10]. New studies showed that seed nano-priming is able to activate different genes during the germination, especially those related to plant stress resistance [14,27,28,29]. The use of nanotechnology for seed priming is a new area of research, although studies have already shown promising results [14,18,29,30,31]. Seed nano-priming also can be used for seed protection, as many nanoparticles have antimicrobial activities and also can load antimicrobial agents [18,32]. In addition, nano-priming can be used aiming biofortification of seeds to promote an increase in food quality and production [1,10,18]. A summary of the main topics that will be covered in this review is shown in Figure 1, as well as the potential applications of seed nano-priming in agriculture.

The aim of this review is to provide an update concerning the potential applications of seed nano-priming techniques in agriculture. Many different types of nanoparticles can be used, with effects on seed metabolism and on the subsequent plant growth and development.

## 2. Seed Priming and Nanoparticles: Definition and Potential Applications

### 2.1. Germination and Principles of Seed Priming

Germination is one of the most important steps for the establishment of plants in agriculture and is fundamental for crop quality [12,18,33]. The rapid development of seedlings ensures fast expansion of the leaves and elongation of the roots, which favor the uptake of nutrients, their translocation through the transpiration flow, and biomass production [12,34,35]. Slow germination can expose the young seedling, which is one of the most vulnerable stages of plant life cycle, to many environmental stress conditions or pathogens, resulting in decreases in vigor and crop productivity, leading to economic losses for farmers [19].

**Figure 2:**
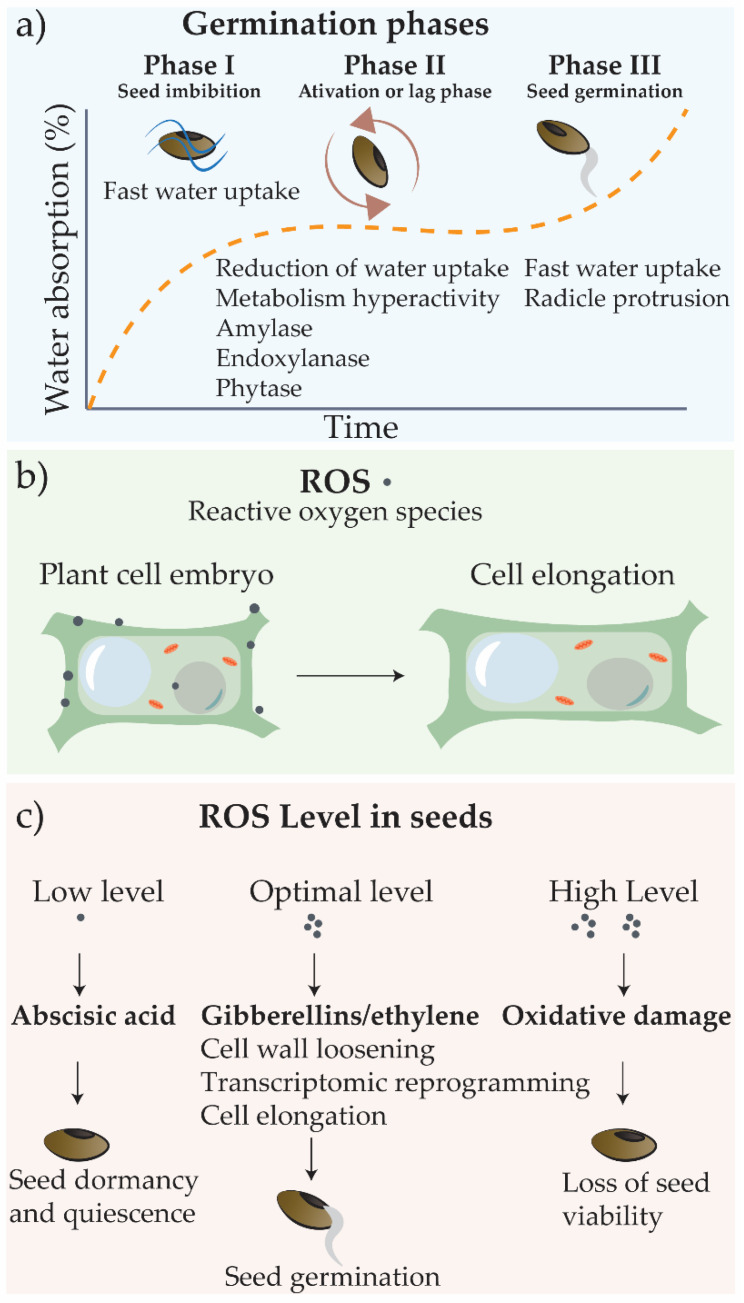
Germination phases and reactive oxygen species (ROS) effects. (**a**) The germination process is subdivided into three phases: phase I (water uptake), phase II (metabolic activity, with initiation of degradation of starch reserves and preparation for embryo development), and phase III (embryo development and emergence of the radicle). (**b**) The ROS act by destabilizing cell wall, allowing water uptake and cell elongation; (**c**) ROS level in seeds and hormones production.

The process of seed germination is usually divided into three phases, as summarized in Figure 2a [36,37]. It begins with imbibition (phase I), when the fast water uptake triggers seed basal metabolism, as transcription, protein synthesis, and mitochondrial activity. In phase II (activation or lag phase), water uptake is limited, but the metabolism becomes hyperactive with the production of enzymes required for reserve mobilization and embryo development, including amylases, endoxylanase, and phytase. In phase III, the seeds exhibit again fast water uptake, and embryo growth culminates in radicle protrusion [36,38].

The seed germination process is finely regulated by signaling molecules that include reactive oxygens species (ROS) and phytohormones [39,40,41] (Figure 2b,c). Apoplastic ROS generation is directly related to cell wall loosening, which allows water uptake and cell extension [39,41]. The abscisic acid and gibberellins work antagonistically to determine seed germination or dormancy, and auxins could also act in the maintenance of seed dormancy [42]. The ROS produced regulate gene expression and phytohormone signaling and the homeostasis of abscisic acid, gibberellins, auxins, and ethylene to control cellular events related to seed germination [40,41,43]. However, when ROS levels are too high, extensive oxidative damages occur, hampering seed germination [41]. ROS content must be, therefore, spatio-temporally controlled to be enclosed in the so-called oxidative window, which ensures proper germination completion [41].

Seed priming is a traditional technique used in agriculture to promote seed germination and plant establishment, based on a preliminary preparation of seeds prior to sowing [18,38,44,45,46,47] It is usually a water-based methodology, whereby seeds are hydrated followed by drying, or some physical methods are applied as ultraviolet light (UV) priming [48]. The absorption of water must be adequate to trigger the metabolic pathways for pre-germination (phases I and II), without resulting in radicle emergence (Figure 2).

This process affects the seed metabolism at molecular and cellular levels (e.g., transcriptomic reprogramming, enhanced capacity for reserve mobilization, cell wall loosening, increased potential for protein synthesis and post-translational modifications), inducing a particular physiological state, which speeds up or even improves the germination and vigor of the primed seeds on a novel imbibition [38,44,45,46,47]. The moderate stress imposed by both soaking and subsequent drying may also induce stress-related responses (e.g., antioxidant mechanisms, heat-shock proteins), resulting in cross-resistance to other stress factors. Moreover, a faster germination shortens per se the exposition of the germinating seeds to adverse soil conditions. Thus, seed priming has been successfully used to accelerate and synchronize germination, improve seedling vigor, and make the plants more resistant to abiotic and biotic stresses, resulting in improvements of productivity and food quality [19,34,47].

Different types of seed priming can be used, such as hydro-priming or hydro-thermo-priming, where the seeds are hydrated using water treatment (usually limited to period of 7–14 h), which allows the occurrence of germination phase II [48]. The technique may be applied with temperature alternation (cold and hot) [49]. In the case of osmo-priming, solutions of low water potential are used to control hydration (around 10–20%), altering seed metabolism through an additional abiotic stress factor [48]. Other methodologies, such as halopriming, hormo-priming, and bio-priming, can be used as pre-sowing treatments, using solutions containing salts [50], plant growth regulators [51], and microorganisms [48], respectively.

### 2.2. Nanoparticles for Seed Priming

Seed nano-priming is a new technology that uses nanomaterials, mainly nanoparticles, for seed priming [14,31,52]. There is an important difference between seed priming and seed nano-priming, since conventional seed priming mainly employs water (hydropriming) or solutions containing substances (nutrients, hormones, or biopolymers) that adsorb on the seed and can result in seed coating (or dressing). In seed nano-priming, the media used are suspensions or nanoformulations, where the nanoparticles may or may not be taken up by the seeds [19]. Even when nanoparticle uptake occurs, the greatest fraction is retained on the seed surface as coating [33,53,54,55]. Such seed coating can be used with fungicides or bactericides in order to protect against pathogens in the field or during storage [56].

One of the first studies showing the potential of nanomaterials to affect seed germination was reported by Khodakovskaya et al. [57]. Although seed priming was not used, these authors demonstrated that carbon nanotubes could be taken up by tomato seeds. The carbon nanotubes increased water uptake, resulting in tomato plants with a 2-fold higher number of flowers [58]. Other studies have similarly demonstrated that carbon nanotubes can modulate seed metabolism in plants, such as tomato, barley, soybean, and maize, increasing the gene expression of several types of water channel proteins [59,60].

Different nanomaterials, including metallic, biogenic metallic, and polymeric nanoparticles, have also shown potential for seed nano-priming [14,54,61], resulting in the stimulation of plant growth and improvement in morphological and metabolic traits. This process can promote fast root and shoot development, with changes in the expression of genes that modulate metabolic processes, such as phytohormone production. Seed nano-priming changes the activity of the defense system, increasing the antioxidant levels and enzyme activities, so that the plants become more resistant to pests and other biotic and abiotic stresses under field conditions [12,31,53,62,63,64].

### 2.3. Active Nanoparticles and Nanocarriers Systems

**Figure 3:**
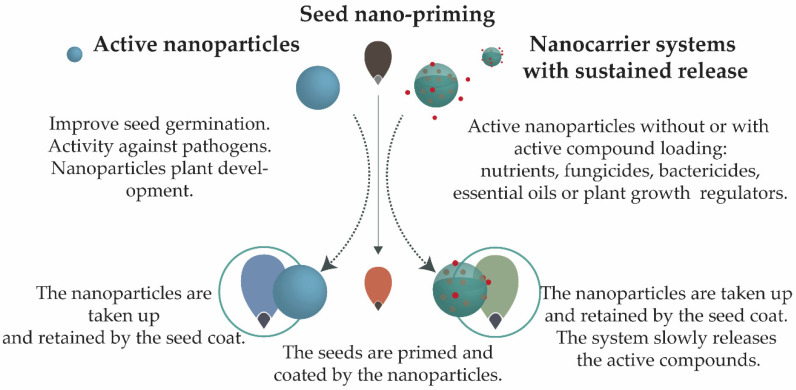
Seed nano-priming. The nanoparticles can be defined as (i) active nanoparticles, which are systems that can have a stimulatory effect on plant growth and development, or (ii) nanocarrier systems providing sustained release, where the nanoparticles can be active or inactive, and are loaded with an active ingredient.

The potential applications of nanoparticles will be considered in two groups: (i) active nanoparticles and (ii) sustained release nanocarrier systems (Figure 3). Table 1 shows systems that have been employed for seed priming/coating, together with their potential effects as stimulants or against biotic and abiotic stress.

Active nanoparticles are nanoparticles that themselves can cause a biological effect, acting as a stimulant, an anti-pathogen, or both [14,61,66]. Nanocarriers with sustained release features are systems where the nanoparticle (being itself active or not) is loaded with an active ingredient (biological or synthetic) and provides extended release of this compound over time [8,9,54,88].

Metallic nanoparticles are an example of active nanoparticles that can be prepared employing either chemical or biological synthesis [14,31,65]. Polymeric nanoparticles are other potentially active systems, since many of the polymers used for their preparation present biological activity [62,70,73]. These systems can be used for sustained release after being loaded with substances, including pesticides, fertilizers, biological compounds, or even other nanoparticles. The sustained release of these substances can result in higher biological activity and reduced toxic effects [54,78,89]. Many products that are used for seed priming can be loaded into nanocarrier systems, consequently improving their biological activities [54,78,82].

#### 2.3.1. Active Nanoparticles

Metallic nanoparticles, including those produced using biogenic processes, are systems that are normally smaller than 100 nm. These systems have received special attention for seed nano-priming, since many of them are prepared with metals that play important roles in plant metabolism and plants biofortification [1,77].

Some examples of these metals are iron, zinc, and manganese. Iron is a co-factor for enzymes, such as cytochrome P450s and Fe(II)/2-oxoglutarate-dependent oxygenase, which makes it an essential nutrient in various metabolic pathways, including respiration and photosynthesis [31,75]. Zinc is another metal essential for plant metabolism. Around 30% of the global lands is deficient in this micronutrient, leading to problems in sugar production and the synthesis of cell membranes, hormones, and proteins, affecting seedling vigor, photosynthesis, and plant defense systems [33,64]. Manganese acts as a co-factor for enzymes as superoxide dismutase (involved in antioxidant response) and the oxygen-evolving complex of photosystem II [29].

Seed nano-priming using nanoparticles based on these metals has shown considerable potential for agricultural applications. For example, Dileep Kumar et al. [67] primed Chilli seeds with metal oxide nanoparticles (zinc, titanium, and silver) and found that zinc oxide nanoparticles improve germination and seedling development (shoot and root length), while these effects are not observed for the other tested metal-based nanoparticles (titanium and silver). According to Rizwan et al. [66], seed priming with zinc oxide and iron oxide nanoparticles improved plant development at high concentrations, increasing spike length, plant biomass, chlorophyll contents, and photosynthetic parameters in leaves. Moreover, the plants presented high concentrations of Zn and Fe after seed priming. Maswada et al. [65] reported an increase in seed germination and plant growth in sorghum treated with iron oxide nanoparticles. Treatment at 500 mg/mL increased photosynthetic pigments and biomass. Treatments at 100 and 500 mg/mL increased the relative water content in leaves.

Duran et al. [53] showed that copper nanoparticles had different effects in bean plants according the nanoparticle size and concentration, with low concentrations and larger nanoparticles resulting in a higher biomass, while high concentrations inhibited seed germination, independently of nanoparticle size.

Biogenic nanoparticles prepared using biological extracts from plants, fungi, or bacteria can have high contents of phytochemicals compounds, including phytomolecules rich in hydroxyl and carbonyl functional groups, such as phenolics, flavonoids, terpenoids, sugars, and proteins. These compounds are natural reducing agents for metals and can act as capping that stabilizes the nanoparticles in colloidal solutions [34].

As an alternative methodology for the synthesis of metallic nanoparticles, green synthesis is more economical compared to chemical methods and has the advantages of increasing biocompatibility and avoiding the use of toxic chemicals [19]. Many biogenic metallic nanoparticles have been shown to present improved biological effects, as well as lower (or no) phytotoxicity, compared to metallic nanoparticles synthesized using chemical methods [64,72].

Some metallic nanoparticles can become toxic to seeds or plants due to the re-oxidation process. For example, silver nanoparticles can be re-oxidized (from Ag^0^ to Ag^+^ ions) within seeds and plants, and the ionic form can inactivate proteins and enzymes, resulting in toxic effects [72]. However, bioactive compounds present as coatings on biogenic nanoparticles can act as stabilization agents, avoiding the re-oxidation reaction and consequently increasing the biocompatibility of metallic nanoparticles [72,83]. Different biogenic metallic nanoparticles have been shown to be able to enhance seed germination, improve plant growth and development, and increase the levels of chlorophyll and antioxidants [12,31,34,77].

Itroutwar et al. [64] found that biogenic zinc nanoparticles produced from brown seaweed (*Turbinaria ornata*) extracts increased seed germination, vigor index, and seedling development. In addition, the plants showed high contents of antioxidant enzymes, and there was a dose-response accumulation of zinc in the seedlings, according to the concentration used for seed priming. Mahakham et al. [34] primed aged maize seeds with biogenic gold nanoparticles synthesized using galangal rhizome extracts. They showed that the treatment at 10 ppm improved the emergence from aged seeds by 83% and seed vigor index by 3-fold, compared to the control, and increased chlorophyll content. Mahakham et al. [14] found that priming rice seeds with biogenic silver nanoparticles produced using kaffir lime leaf extract increased the α-amylase activity and water uptake of the seeds, improving both germination and plant biomass [15]. Kasote et al. [31] primed watermelon seeds with biogenic iron nanoparticles produced using onion extracts, which resulted in the increase of germination and growth of shoots and roots. The nanoparticles did not cause adverse antioxidant and chlorophyll effects, compared to the effects of the bulk counterparts (FeCl_3_ and Fe_2_O_3_). The nanoparticles were taken up and translocated into the seed endosperm, leading to increased non-enzymatic antioxidant levels and the induction of jasmonates-linked defense responses in the seeds.

Acharya et al. [19] performed, for 2 years, priming onion seeds with biogenic silver and gold nanoparticles produced using onion extract. The treatments resulted in increase of seedling emergence, number of leaves, plant weight, and productivity, compared to unprimed and hydro-primed seeds. The results also showed metabolic effects, with increases of chlorophylls *a* and *b*. The gold nanoparticles provided better results than the silver nanoparticles.

Biogenic metallic nanoparticles can act as delivery systems, since many secondary metabolites may adhere on the nanoparticle surface. These metabolites can have different biological actions that improve plant metabolism and growth [64,90]. In addition, many biological extracts have fungicidal or bactericidal properties and can be used in the protection against pathogens [91,92]. Furthermore, metallic or biogenic metallic nanoparticles can be taken up by seeds more easily due their small sizes and trigger secondary metabolites more efficiently, providing stronger action in development of the embryo [14,31,35].

#### 2.3.2. Sustained Release Nanocarrier Systems

Biopolymeric nanoparticles are systems that can be made from polysaccharides, lipids, and proteins, resulting in nanoparticles that are biodegradable and biocompatible, besides being able to be designed to respond to different environmental stimuli [6,8,9]. These systems can be loaded with various substances, including fungicides [93,94], essential oils [89,95,96], plant growth regulators [54,97,98], and fertilizers [99].

Polymeric nanoparticles used for seed coatings are normally larger than 100 nm [54], providing slow release of active compounds used to modify plant metabolism or to combat pathogens [9,54,81,88]. Numerous nanocarrier systems based on biopolymers have the potential to be used for seed treatment, including alginate [100,101], zein [89,102], cellulose [103,104], synthetic biopolymers (poly-epsilon-caprolactone, poly(lactic-co-glycolic acid), poly(lactic acid)) [9], and lipid nanoparticles [105,106].

Chitosan is another biopolymer that has been used to prepare nanocarrier systems for agricultural applications. This polymer has fungicidal properties and also acts in plant metabolism, activating defense mechanisms [94,107,108]. The concentration of this biopolymer is a key consideration, since high concentrations block seed germination [73]. This effect is because oligosaccharides and polysaccharides can elicit physiological responses in plants, high concentrations causing cell apoptosis, while low concentrations have stimulant effects [62].

Biopolymeric nanoparticles can be used as carrier systems for metal micronutrients, allowing a slow release of these compounds. Saharan et al. [85] showed that the priming of maize seeds with chitosan nanoparticles containing copper induced α-amylase and protease activities and promoted reserve mobilization, thus favoring seedling growth. In contrast, the treatment with bulk copper led to inhibitory effects. Similarly the priming with zinc-loaded chitosan nanoparticles induced the activity of hydrolytic enzymes in maize seeds, favoring germination.

Nanocarrier systems for plant growth regulators have shown potential for seed priming, providing improvements from seed germination up to production [54,82]. Falsini et al. [54] used lignin nanoparticles containing gibberellic acid to coat arugula and tomato seeds. The effects varied according to the time of sowing after the treatment. In arugula seeds, the formulations improved germination and increased the stem and root lengths and the plant biomass. Similar effects were observed for tomato seeds, varying according to the parameter and the concentration used. The ability of active nanoparticles or nanocarrier systems to increase seed germination can be explained by the fact that, at optimal concentrations, nanoparticles are able to penetrate the seed coat, increasing the number of holes in the coat and, consequently, increasing water uptake and oxygen transfer [58,75,76].

In another work with tomato seed priming, Pereira et al. [82] reported that chitosan-based nanocarrier systems of gibberellic acid were able to improve not only initial seedling growth but also fruit production under field conditions^.^ In this study, the seed nano-priming was able to increase the production in 225.5% and 178.8% for chitosan/tripolyphosphate and alginate/chitosan nanoparticles containing gibberellic acid, respectively. The methods used to prepare these nanoparticles do not involve organic solvents, being interesting method aiming a sustainable agriculture [97,109].

Muthuhrishnan et al. [81] used chitosan nanoparticles loaded with thiamine to prime chickpea seeds. This procedure improved seed germination, induced a 10-fold increase of auxin level in seedlings, and boosted the quantity of secondary roots.

The improvement of the biological efficacy of active compounds by nanocarrier systems is associated to the enhancement of physical-chemical proprieties. Effects, such as increased solubility, protection against degradation, and greater bioavailability, can reduce the required concentration of the active compound and minimize toxic effects [6,110]. Several studies have shown that seed-nano priming can be more efficient than conventional methods and can contribute to improved agricultural practices [14,19,78]. Many systems defined as nanopesticides or nanofertilizers, used in the control of pests or for plant development, have the potential for use in seed nano-priming [3,8,111].

### 2.4. Seed Nano-Priming and Effects on Plant Metabolism under Abiotic and Biotic Stresses

Abiotic and biotic stresses lead to reduced production and economic losses. Abiotic stresses are environmental factors, such as drought, flood, heat or cold, salinity, or nutrient-deficient soils. Biotic stresses are caused by microbial pathogens (bacteria or fungi), insects, or weeds that compete for nutrients [4,8].

The use of seed nano-priming in agriculture can improve the quality of seeds and increase resistance against stress conditions. Nanoparticles can act directly against pathogens, as well as alter the metabolism of seeds and plants, consequently enhancing the innate immune system, altering hormone production, and making the plants more resistant to diseases or abiotic stress [112].

**Figure 4:**
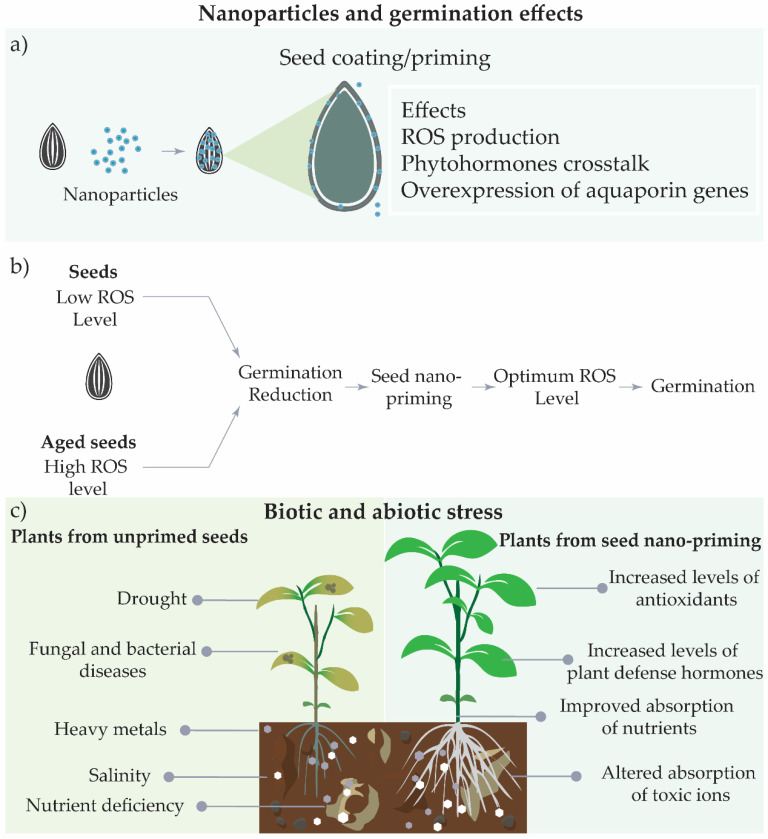
Effects of seed nano-priming on seeds and plants under abiotic and biotic stresses. (**a**) Effects of seed nano-priming during the germination, (**b**) effects of seed nano-priming on ROS levels, and (**c**) potential effects under biotic and abiotic stresses.

The nanoparticles uptake under seed coating can promote the ROS production, acting in different metabolic pathways, increase the level of active gibberellins, and the mobilization of storage proteins [35]. In addition, the effect of nanoparticles in increasing water uptake by the seeds can cause sufficient stress to activate germination, increasing the activities of enzymes in phases I and II of the process [76] (Figure 4a). Seed nano-priming has been shown to increase germination, since these systems are able to keep ROS levels in the optimum range comprised by the oxidative window that promotes seed germination (Figure 4b) [31,75].

Under stress conditions, nanoparticles can act to reduce seed ROS levels, due to increased activity of enzymes, such as superoxidase dismutase, catalases, and guaiacol-peroxidase, hence reducing seed cell damage [75].

The storage of seeds for long periods at low temperatures results in aging, which can heavily decrease the germination rate [34]. After long periods, the seed cells initiate processes that increase ROS generation and reduce antioxidant levels, causing metabolic side effects that result in a reduction of the germination index [75].

The use of biogenic metallic nanoparticles has been shown to be able to ROS at optimum levels, resulting in improved germination of aged seeds. The biogenic nanoparticles can be coated with many compounds that are natural reducing agents and act to decrease ROS levels in seeds [14,75].

#### 2.4.1. Biotic Stress

Seed protection is required at different times during agricultural practices. In the field, the seeds and seedlings need to be protected against insects, fungi, and bacteria that can damage the seed and reduce germination or seedling growth. During harvesting and storage, seeds carry microorganisms from the field. These may shorten the shelf-life of the product or lead to the accumulation of microbial metabolites that make the products dangerous for consumption [113].

In the field, the use of fungicides and neonicotinoids for seed coating can reduce the damage caused by these organisms during plant establishment [56]. This method has shown excellent potential compared to foliar applications [114,115]. Relative to foliar treatment, the use of pesticides for seed treatment can reduce the amounts of the chemicals applied in the field, as well as decrease the number of runs of agricultural machines on the land (avoiding soil compaction); so, the technique can be considered economically and environmentally advantageous [114]. The technology of seed treatment in developing countries is expected to reach a gain of US$ 1.63 billion [32].

Seed coating techniques employ fungicides that may be released in the field during the sowing process, resulting in environmental contamination. For example, pesticides, such as carbofuran and thiran, are highly toxic and persistent in the environment [11], so the consumption of treated seeds by animals, such as granivorous birds, can lead to poisoning or death [56].

Because nanoparticles are reactive and physiochemically dynamic materials, they tend to heteroaggregate in environmental media [116]. This can result in an effective seed coating, limiting the release of these compounds in the environment [116].

Nonetheless, the use of nanoparticles for seed priming and coating can provide eco-friendly solutions. Many of these systems are nontoxic, biodegradable, and can increase the bioefficacy of active ingredients used at low concentrations [14,73,78].

Several systems have been shown to be effective against pathogens. Ahuja et al. [68] demonstrated that iron(II) sulfide nanoparticles were more effective than the fungicide carbendazim to control the fungus *Fusarium verticillioides* in rice seeds. Silver nanoparticles are active against plant pathogens, including *Aspergillus flavus*, *Aspergillus niger*, *Aspergillus fumigatus*, and *Colletrotrichum capsica*, showing great potential for seed protection. Metallic nanoparticle systems can be used for both seed priming and seed protection, as demonstrated for nanoparticles composed of iron [68], copper [117], silver [14], and silica [78]. Sathiyabama and Muthukumar [73] primed rice seeds with chitosan/guar nanoparticles, which resulted in increased plant development and higher levels of chlorophyll and carotenoids, the nanoparticles showing anti-fungal activity (71%) against the rice pathogen *Pyricularia grisea*. For pearl millet, Nandhini et al. [113] showed that seed nano-priming and foliar application of zinc nanoparticles reduced downy mildew caused by *Sclerospora graminicola*. Dileep Kumar et al. [67] showed that nanoparticles of zinc, titanium, and silver could reduce pathogen infection as *Aspergillus flavus*, *Aspergillus niger*, *Aspergillus fumigatus*, and *Colletrotrichum capsica* in Chilli seeds.

Siddaiah et al. [61] showed that the treatment of millet seeds with chitosan nanoparticles resulted in alteration of the innate immune system of the plants and increased resistance against pathogens. The treatment improved the levels of antioxidant enzymes and increased expression of proteins involved in the salicylic acid pathway, which is related to plant resistance responses against biotrophic pathogens. The field studies of Choudhary et al. [86] showed that the treatment of maize seeds with zinc-containing chitosan nanoparticles resulted in plants with higher activity of antioxidant enzymes and lignin concentrations, making the plants more resistant to pathogens. Bravo Cadena et al. [78] showed that silica nanoparticles loaded with cinnamon essential oil and applied to pea seeds acted as seed primers and provided bactericidal activity against *Pseudomonas syringae* pv. *pisi*, even at low concentrations. The seeds showed fast germination, and the cinnamon essential oil provided a 90,000-fold increase in bactericidal activity in comparison to the non-encapsulated oil. The use of essential oils with fungicidal or bactericidal properties for seed priming is a promising eco-friendly alternative to chemical fungicides or bactericides [78,86,89].

Techniques employed during the storage of cereals include the control of humidity and temperature to minimize the growth of microorganisms, as well as the use of chemical products, although the latter may be viewed negatively by consumers, who prefer natural products [113]. For this purpose, the use of biopolymers or essential oils with activities against pests can provide a greener alternative to agrochemicals, avoiding problems associated with residual contamination.

#### 2.4.2. Abiotic Stress

Harvest yields can be drastically decreased by soil salinity and contamination [65]. Salinity represses plant growth due to water and nutritional deficits and direct ionic effects on plant metabolism [63]. Contamination with heavy metals may be both anthropogenic (due to fertilizer applications, industrial processes, atmospheric deposition following waste incineration, and discharges of sewage and sludge) and geogenic (due to natural atmospheric deposition or regional geological processes) [30,118,119].

Ye et al. [29] showed that jalapeño pepper seeds (*Capsicum annuum* L.) primed with manganese nanoparticles presented improved germination and root elongation under saline conditions, as well as alleviated salt stress by modulating successfully the distribution of sodium between roots and shoots, through control of the oxidative stress.

Abdel-Latef et al. [63] demonstrated that lupin seeds primed with zinc nanoparticles mitigated the salinity stress condition, avoiding reductions of growth parameters (root and shoot lengths; fresh and dry weights) and photosynthetic pigments.

Maswada et al. [65] reported that sorghum seeds treated with nano-iron(III) showed improved germination, with the plants showing increased chlorophyll contents and improved growth under saline conditions. These results indicate that this system could be used not only to improve seed germination but also to avoid stress conditions. In another study, priming lupin seeds with zinc nanoparticles improved plant development under saline conditions, due to increased levels of photosynthetic pigments, phenols, organic molecules, and antioxidant enzymes.

In a case of heavy metal contamination, Rizwan et al. [66] showed that priming of wheat seeds with iron and zinc nanoparticles reduced the absorption of cadmium, resulting in low cadmium concentrations in the grains. Zinc nanoparticles reduced cadmium levels in shoots, roots, and grains by 38%, 55%, and 83%, respectively. The iron nanoparticles reduced cadmium levels in the shoots, roots, and grains by 54%, 56%, and 84%, respectively. The plants presented high concentrations of zinc and iron after seed priming [68]. Hussain et al. [30] demonstrated that, when germinated in soil contaminated with cadmium, seed priming with silicon nanoparticles was able to reduce cadmium uptake, to increase plant biomass, photosynthetic rate, and levels of carotenoids and chlorophylls *a* and *b*, and to decrease the formation of reactive oxygen species and antioxidant enzymes activity.

Soil deficiency of nutrients, such as iron and zinc, can affect the production of phytohormones involved in plant defense responses, including jasmonic acid and salicylic acid [31,86]. Kasote et al. [31] primed watermelon seeds with biogenic iron nanoparticles prepared using onion extract. The plants showed increased levels of jasmonic acid and of its precursor cis-(+)-12-oxo-phytodienoic acid during the early seedling stage, which could increase the resistance to stress.

Nguyen et al. [117] showed that maize seed priming with cooper nanoparticles can enhance drought resistance in plants. The leaves kept high water content, increased the levels of anthocyanin, chlorophyll, and carotenoids, and reduced the oxidative stress.

These results showed the potential of seed-nano priming to relieve plant stress caused by saline conditions, drought, the presence of heavy metals, or nutrient deficiency, with the modulation of metabolism to resist stress conditions and improve plant growth (Figure 4c). The use of seed nano-priming to trigger plant resistance to abiotic stress is a strong alternative to avoid side effects of global climatic crisis or anthropogenic and geogenic effects, in order to reduce losses in the field production.

### 2.5. Molecular Responses Induced by Seed Priming During Germination, Abiotic and Biotic Stress

Nanoparticles are able to interact with plant cells and to be internalized in different cell compartments. Seed nano-priming can initiate or alter many genes expression profiles and biochemical pathways [28,35] during the germination step and even over time [14,27].

Water exchange during the imbibition phase is the first step of seed germination. During this germination process, seed priming with nanoparticles can induce the expression of aquaporin genes, increasing water uptake in seeds [14,59]. Multiwalled carbon nanotubes air-sprayed on seeds of crops (barley, corn, and soybean) activated the expression of seed-located water channel genes (aquaporins) from different subfamilies [59]. According to Mahakham et al. [14], aquaporin genes were overexpressed in rice seeds (*Oriza sativa* L. cv. KDML 105) primed with silver nanoparticles. Aquaporins allow the diffusion of water across biological membranes and facilitate the transport of gases (CO_2_, NH_3_), nutrients, and ROS (especially H_2_O_2_). Thus, together with the induction of aquaporin genes triggered by seed nano-priming, the authors reported a faster germination than in controls.

An et al. [27] used transcriptomic analyses of cotton seeds primed with cerium oxide nanoparticles coated with the antioxidant poly(acrylic acid) and exposed or not to a saline stress. Seeds treated with nanoparticles, under no saline stress, resulted in the expression of 7799 different genes in comparison to the control. In salinity conditions, the authors described that the nano-primed seeds expressed 13 genes related to ROS pathways and 10 genes related to ion homeostasis.

In another example, Ye et al. [29] showed that manganese nanoparticles used for seed priming of *Capsicum annuum* L. also promoted the seedling growth under saline conditions. In this study, the primed seeds up-regulated *MnSOD* gene (*Mn superoxide dismutase*), increasing SOD enzyme levels, which defends plant cells against ROS damage, avoiding the phytotoxic effects.

The seeds’ nano-priming treatment also demonstrated to regulate resistance genes against biotic stress. Siddaiah et al. [61], showed that seeds treated with chitosan nanoparticles increased the resistance against downy mildew disease caused by the biotrophic oomycete *Sclerospora graminicola*. The seed priming improved the innate immune system. The plants increased the expression of genes of phenylalanine ammonia lyase, peroxidase, and polyphenoloxidase. Moreover, the plants overexpressed pathogenesis-related (PR) genes (*PR1* and *PR5*), that are involved in the salicylic acid pathways.

Plaksenkova et al. [120] reported that barley seeds exposed to zinc oxide nanoparticles increased the expression of the microRNAs miR156 and miR159, that are involved in the plant mechanisms against abiotic and biotic stress.

In summary, the effects of seed nano-priming at molecular level and how nanoparticles modulate gene expression are not totally elucidated, and research in this field is extremely important. The different kinds of nanoparticles, treatments, and concentrations can result in different responses in plant metabolism. In addition, nanoparticles can act as a signal or co-factors improving the regulation of transcription of genes related to phytohormones and response to biotic and abiotic stress conditions [29,35].

### 2.6. Effects on Microbiota

The effect of seed nano-priming on plant microbiota is a fairly new area requiring further studies. The interaction between plants and microorganisms is essential for agriculture, since these symbiotic relations are important for the absorption of nutrients, defense against pathogens, plant quality, and productivity [87].

The foliar application of nanoparticles can modulate the root microbial community. Raliya et al. [121], showed that foliar application of zinc nanoparticles to mung beans led to an extension of the rhizosphere zone, with root volume increasing by 58.9%. They also reported increases in the activity of rhizosphere enzymes, such as acid phosphatase (98.07%), alkaline phosphatase (93.02%), and phytase (108%). These factors are extremely important for the uptake of nitrogen and phosphorus by plants.

Dai et al. [122] found that roots exposed to cerium oxide nanoparticles decreased the rhizosphere bacterial community but enhanced the growth of microorganisms that promote plant growth.

Other studies have reported side effects in the soil microbiota following amendments with nanoparticles. Zhang et al. [123] found that silver nanoparticles (at 100 mg/kg) altered the soil pH and negatively affected microorganisms involved in nitrogen, carbon, and phosphorus cycles. In another study, Li et al. [124] showed that the amendment of soils with silver nanoparticles (10.4 mg/kg) reduced plant development and resulted in silver bioaccumulation.

Other metallic nanoparticles, such as titanium and zinc nanoparticles, have been found to affect the soil microbiota, depending on the concentration and the duration of exposure [125,126]. Apart from the intrinsic properties (chemical nature, size, coating) and concentration, the impact of nanoparticles on plant root and rhizosphere microbiota are largely dependent on soil texture, pH, organic matter content, and fertilization [127,128,129].

Few studies have investigated how seed nano-priming might affect the interactions of microorganisms with roots. Rahman et al. [71] showed that seed priming using platinum nanoparticles stabilized with poly(vinylpyrrolidone) resulted in side effects on the root microbiota of pea plants, with a decrease in mycorrhizal fungi and rhizobial colonization, while these effects were not observed for the same system with gold or silver nanoparticles.

More studies in this field must be encouraged in order to elucidate the mechanisms by which seed-nano priming can affect seed metabolism and alter the microbiome selected by the roots during development of the plant. Many nanoparticle systems have shown great potential for use in agriculture but could be toxic towards soil microbiota. The treatment of seeds may offer a safer way to improve the establishment of plants and avoid side effects in the soil, since the treatment is performed on the seeds, avoiding applications in soils.

### 2.7. Improving Crop Quality and Production

Seed nano-priming can increase the productivity of different crop species, due to the positive effects on plant metabolism and development. Fast root development increases the potential of the plant to access nutrients and water, accompanied by faster expansion of the leaf area, consequently increasing the use of light energy for plant growth.

The effects on the innate immune system can improve the resistance against pathogens, so that smaller quantities of pesticides need to be applied. Consequently, the concentrations of residual agrochemicals in food are lower, making the products safer for consumption. These effects have been demonstrated in studies evaluating the effects of seed priming, up to harvest, in species with agronomical value.

Yasmeen et al. [79] showed that, in wheat plants, seed priming with iron and copper nanoparticles led to improvements of spike length, number of grains per spike, and grain weight. Rahman et al. [71] reported that priming of pea seeds with platinum nanoparticles stabilized with poly(vinylpyrrolidone) increased production by 163.5%, although the seed weight was reduced by 66.7%.

Acharya et al. [12]) demonstrated the potential benefits of priming watermelon seeds with biogenic silver nanoparticles produced using onion extracts. The results revealed a burst in plant development and increased metabolic activity throughout the life of the plant, resulting in production increases between 31.6% and 35.6%.

Pereira et al. [82] reported that the priming of tomato seeds with alginate/chitosan nanoparticles containing gibberellic acid greatly improved fruit production, increasing productivity by almost 4-fold.

Joshi et al. [76] showed that wheat seeds treated with multi-walled carbon nanotubes resulted in the increases of seed germination, root length, number of root hairs, shoot length, plant weight, number of stomata, and size of average length of vascular systems (xylem and phloem). At 90 µg/mL, there were increases of 21% and 27% for spike length and weight, respectively, 20% for the number of spikelets, and 32% for grain production.

Another issue concerns biofortification. For example, the treatment of seeds with iron, zinc, and manganese nanoparticles resulted in plants and grains with higher contents of these minerals [74]. Other studies have also reported that increased concentrations of these elements in roots, shoots, leaves, or grains can result in vegetables richer in essential minerals [66,74].

Seed nano-priming can be an effective way to reduce the amounts of fertilizers applied to crops. A major problem in agriculture is that only 30–50% of the nitrogen and 45% of the phosphorus applied in the field are absorbed by the crops [77]. These losses can be harmful to the environment, since they can lead to the eutrophication of aquatic systems and contamination of native terrestrial ecosystems.

Das et al. [77] in fields studies showed that rice seeds primed with nano-pyrite (FeS_2_) promote the same productivity found for untreated seeds, with seedlings grown in the presence of fertilizers.

The use of seed nano-priming has been shown to improve root development and increase the production of enzymes required for nutrient uptake, which would enable reductions of the amounts of fertilizer used in the field [75].

## 3. Concerns

Although nanoparticles have potential for use in seed priming or seed coating, caution must be exercised in the application of nanomaterials. The safe use of these systems demands the development of appropriate regulations based on sound research, not only in agriculture, but also in many other industrial sectors [5,130]. Legal frameworks are required for industrial production of nanomaterials, treatment of industrial waste, and agricultural applications, together with evaluation of the fates of these nanoparticles in the environment, considering their possible ecotoxicity.

Agricultural activities are connected with many ecosystems that may be directly impacted by nanomaterials [3]. Therefore, it is crucial to understand the mechanisms of action of these materials and to develop nanoparticles that are safe in both the field and the wider environment.

Before seed treatment, it is necessary to evaluate the conditions employed for priming, considering the nanoparticle size and concentration, and the duration of exposure, since these factors can cause side effects, such as germination inhibition, reduced plant development, detrimental alterations of metabolism and cell structure, and modification of root-microbiota interactions [56,66,71].

It is essential to understand how the physical-chemical properties of nanoparticles affect seeds and other associated organisms, in order to be able to design nanoparticles that are both effective and present minimal toxic effects [8].

The different kinds of nanoparticles cited in this article (metallic, biogenic metallic, and polymeric nanoparticles) not only differ in their physical chemical characteristics, but also in their biological activity. The design of these nanoparticles for seed priming can be used for different strategies, as example seed protection, biofortification, plant resistance against pests and abiotic stresses, or even the mix of these effects.

However, the use of nanoparticles for seed priming can provide great advantages. The treatments in seeds reduce the exposition of nanomaterials compared with foliar and soil applications. Another positive point is the low concentrations of nanoparticles used for seed priming, that can be a through a controlled form by factories, avoiding high release of this materials in the environment. Probably, the residual of nanoparticles in plants will be very small or even none, but studies are necessary to elucidate how different nanoparticles as metallics, metallics biogenic, and polymerics interact with the plant development.

## 4. Conclusions

Nanotechnology is a promising area for exploitation in agriculture, and seed-nano priming is one of the tools that can be employed to promote sustainability.

The use of nano-based technology for seed treatment has potential to move the traditional agriculture based on the use of agrochemicals to a more sustainable agriculture, once these systems can promote the establishment of plants, as well as provide protection against biotic and abiotic stresses, resulting in improvements of productivity and food quality. All these factors together can result in a system safer for farmers and consumers, with respect to the environment avoiding the continuous damage caused by the conventional agriculture.

There are many issues that need to be addressed concerning the industrial production of these technological systems and their application in the field, including scale-up, seed priming conditions, and toxic effects in plants and other organisms. However, it is clear that the adoption of nanoparticle systems can alter crop management, with reduction of the applied quantities of pesticides and contamination risks, resulting in agricultural practices that are safer for farmers, consumers, and the environment.

## Figures and Tables

**Table 1:** Nanoparticles employed for seed priming and coating, showing the nanoparticle systems, their characteristics, and the main effects on the species evaluated. Each article is classified according its application: A1 (seed priming), A2 (effects in the field), A3 (effects against pathogens), and A4 (relieving abiotic stresses). The arrows up and down indicated an increase and decrease in activity, respectively.

Nanoparticle System	Characteristics	Main Effects	Applications	Reference
Biogenic silver nanoparticles produced using kaffir lime leaf extracts	Spherical nanoparticles with particle size of 6–26 nm	**Concentrations:** 10 and 20 mg/mL **Species:** Rice seeds (*Oriza sativa* L. cv. KDML 105). **Effects:** Water uptake ↑, Aquaporin gene expression ↑, Enzyme activity ↑, Seed and seedlings vigor ↑ Plant morphology ↑, and biomass ↑	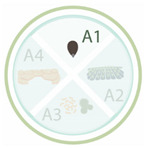	[14]
Iron oxide nanoparticles	Particle size <50 nm, with surface area of 180 m/g^2^	**Concentrations:** 10, 50, 100, and 500 mg/L. **Species:** Sorghum (*Sorghum bicolor* (L.) Moench) KDML 105. **Effects:** Seed and seedlings vigor ↑, Biochemical activity ↑, Biomass ↑, and water content in leaves ↑	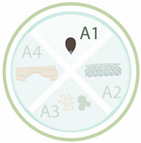	[65]
Biogenic iron nanoparticles produced using onion extracts	Particle size of 19–30 nm, with low-crystalline or amorphous Fe_2_O_3_	**Concentrations:** 20, 40, 80, and 160 mg/L. **Species:** Watermelon (*Citrullus lanatus* (Thunb.) Matsum and Nakay varieties). **Effects:** Seed and seedlings vigor ↑, Plant morphology ↑, Phytotoxic effects ↓. Enzyme activity ↑, and Plant growth regulator (jasmonate) ↑	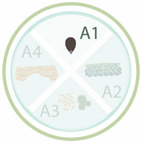	[31]
Zinc oxide and iron oxide nanoparticles	Zinc oxide nanoparticles with sizes of 20–30 cm; iron oxide nanoparticles (Fe_3_O_4_) with sizes of 50–100 nm	**Concentrations:** Zinc nanoparticles at 25, 50, 75, and 100 mg/L; iron nanoparticles at 5, 10, 15, and 20 mg/L. **Species:** Wheat (*Triticum aestivum* L.). **Effects:** Plant morphology ↑, Biomass ↑, Biochemical activity ↑, Cadmium uptake ↓, and Biofortification ↑	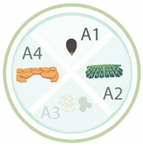	[66]
Silicon nanoparticles	Spherical nanoparticles with size around 90 nm	**Concentrations:** The nanoparticles were evaluated at concentrations of 300, 600, 900, and 1200 mg/L. **Species:** Wheat (*Triticum aestivum* L.). **Effects:** Biomass ↑, Biochemical activity ↑, ROS levels ↑, and Cadmium uptake ↓	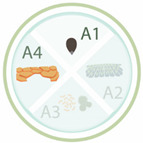	[30]
Biogenic zinc nanoparticles produced using brown seaweed (*Turbinaria ornata*) extracts	Spherical and hexagonal nanoparticles with average size of 15–52 nm	**Concentrations:** Nanoparticles at concentrations of 5, 10, 25, 50, 100, and 200 mg/L. **Species:** Rice (*Oryza sativa* L.). **Effects:** Seed and seedlings vigor ↑, Antioxidant enzymes ↑, and Biofortification ↑	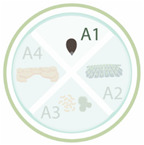	[64]
Nanoparticles of zinc, titanium, and silver	Zinc oxide nanoparticles (35–40 nm), titanium oxide nanoparticles (100 nm), and silver nanoparticles (85 nm), with spherical, cylindrical, and needle-like morphologies, respectively	**Concentrations:** Nanoparticles at concentrations of 750, 1000, and 1250 mg/kg. **Species:** Chilli (*Capsicum annuum* L.). **Effects:** Seed and seedlings vigor ↑, Plant morphology ↑, Antimicrobial activity ↑	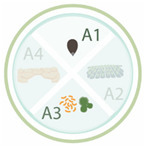	[67]
Chitosan/tripolyphosphate nanoparticles	Nanoparticles with size of 259.4 ± 4.7 nm, PDI (polydispersity index) of 0.28 ± 0.0016, and zeta potential of 40.0 ± 2.9 mV	**Concentrations:** Nanoparticles at concentrations of 1–100 µg/mL. **Species:** Wheat (*Triticum aestivum* L.). **Effects:** Plant morphology ↑, Biochemical activity ↑, and Plant growth regulator (auxin) ↑	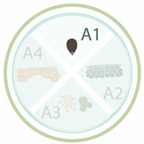	[62]
Biogenic gold nanoparticles synthesized using galangal rhizome extracts	Spherical nanoparticles with size of 10–30 nm	**Concentrations:** Nanoparticles at concentrations of 5, 10, and 15 ppm. **Species:** Maize (*Zea mays* L.). **Effects:** Seed and seedlings vigor (aged seeds) ↑, and Biochemical activity ↑	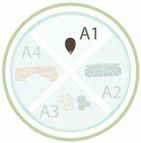	[34]
Manganese(III) oxide nanoparticles	Spherical nanoparticles with size of 50 nm, and zeta potential of 29.2 ± 1.8 mV	**Concentrations:** 0.1, 0.5, and 1 mg/mL. **Species:** Jalapeño (*Capsicum annuum* L.). **Effects:** Salinity resistance ↑, Antioxidant enzymes ↑	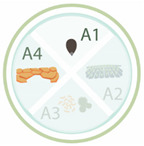	[29]
Iron(II) sulfide aqua nanoparticles	Spherical nanoparticles with size around 6–20 nm	**Concentration:** 30 µg/mL. **Species:** Rice (*Oryza sativa* L.). **Effects:** Seed and seedlings vigor ↑ Plant morphology ↑, Antimicrobial activity ↑	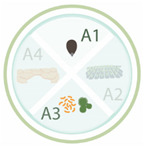	[68]
Copper nanoparticles	Nanoparticles with sizes of 25, 40, and 80 nm, and zeta potentials of 15–25 mV	**Concentrations:** 1, 10, 100, and 1000 mg/L. **Species:** Common bean (*Phaseolus vulgaris* L.). **Effects:** Seed and seedlings vigor ↑↓ (High concentrations inhibited seed germination, independent of nanoparticle size), and Biomass ↑	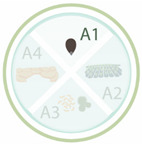	[53]
Chitosan nanoparticles of and carbon nanotubes	Chitosan nanoparticles with size of 95 ± 2 nm and zeta potential of +123.5 mV; carbon nanotubes with size of 40 ± 0.4 nm, and zeta potential of −8.5 mV	**Concentrations:** 10% of both nanomaterials were used, with a concentration of 20 µg/L^−^. **Species:** Common bean (*Phaseolus vulgaris* L.). **Effects:** Plant morphology ↓, ROS levels ↑, and Biochemical activity ↓	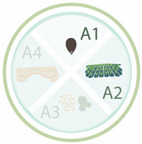	[69]
Zinc nanoparticles	Zinc nanoparticles with size of 20 nm and spherical shape, and sizes of 40 and 60 nm with elongated shapes	**Concentrations:** 1, 10, 100, 1000, and 5000 mg/L. **Species:** Common bean (*Phaseolus vulgaris* L.). **Effects:** Biomass ↑	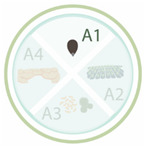	[33]
Chitosan nanoparticles	Chitosan nanoparticles with sizes of 20–170 nm	**Concentrations:** 0.5, 1, 1.5, 10, 15, and 20 mg/mL. **Species:** Rice (*Oryza sativa* L.). **Effects:** Plant morphology ↓, and Biomass ↑	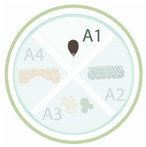	[70]
Zinc nanoparticles	Mean size of 21.3 nm	**Concentrations:** 20, 40, and 60 mg/L. **Species:** Lupin (*Lupinis termis* L.). **Effects:** Salinity resistance ↑, Biochemical activity ↑, ROS levels ↓	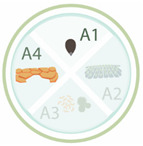	[63]
Biogenic silver and gold nanoparticles produced using onion extract	Silver nanoparticles with size of 11.6 ± 2.40 nm and zeta potential of −2.20 ± 0.29 mV, with spherical and ellipsoidal morphology; gold nanoparticles with size of 93.68 ± 2.06 nm, and zeta potential of −8.51 ± 1.26 mV, with anisotropic morphology	**Concentrations:** Silver nanoparticles at 31.3 µg/mL and gold nanoparticles at 31.3 µg/mL. **Species:** Onion (*Allium cepa* L.) **Effects:** Seed and seedlings vigor ↑ Plant morphology ↑, Biochemical activity ↑	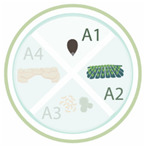	[19]
Platinum nanoparticles stabilized with poly(vinylpyrrolidone)	Nanoparticles with size of 3.2 ± 0.8 nm and spherical morphology	**Concentrations:** Concentrated solution at 1.0 mM. **Species:** Pea (*Pisum sativum* L.). **Effects:** Seed and seedlings vigor ↑ Plant morphology ↑, and microorganisms colonization (arbuscular mycorrhizal fungi and rhizobia) ↓	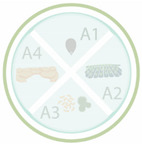	[71]
Biogenic silver nanoparticles produced using *Phyllanthus emblica*	Nanoparticles with size of 10–35 nm, irregular shape, and zeta potential of −23.8 mV	**Concentrations:** 0, 5, 10, 25, and 50 mg/L. **Species:** Wheat seeds (*Triticum aestivum* L.). **Effects:** ROS levels ↓, Seed and seedlings vigor (non-biogenic silver nanoparticles) ↓, Seed and seedlings vigor (biogenic nanoparticles) ↑	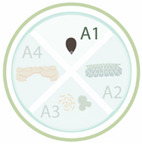	[72]
Chitosan guar nanoparticles	Nanoparticles with size of 122 nm, PDI of 0.358, and zeta potential of −30 mV	**Concentrations:** 0.05, 0.1, and 0.2%. **Species:** Rice (*Oryza sativa* L.). **Effects:** Seed and seedlings vigor ↑↓ (0.05% and 0.1%, the nanoparticles promoted seed germination, while the use of 0.2% reduced seed germination), Plant morphology ↑, Biomass ↑, Biochemical activity ↑ and Antimicrobial activity ↑		[73]
Iron nanoparticles	Nanoparticles with size of ~80 nm, and zeta potential of −44 mV	**Concentrations:** 25, 50, 100, 200, 300, 400, 500, and 1000 µg/mL. **Species:** Wheat (*Triticum aestivum* L.), types WL711 (low-iron genotype) and IITR26 (high-iron genotype). **Effects:** Seed and seedlings vigor ↑↓ (dose dependent), Plant morphology ↑↓ (High concentrations caused inhibition of plant growth), and Harvest ↑	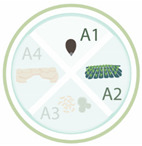	[74]
Zero-valent iron nanoparticles (priming of aged seeds)	Nanoparticles with size of 33.8 ± 3.59 nm, and zeta potential of −39 mV	**Concentrations:** 10, 20, 40, 80, and 160 mg/L. **Species:** Rice (*Oryza sativa* L.). **Effects:** Seed and seedlings vigor ↑↓, Water uptake ↑↓, Plant morphology ↑, Biochemical activity ↑, Enzymatic activity ↑, ROS levels ↓. Dose dependent effects	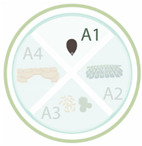	[75]
Biogenic silver nanoparticles and turmeric nanoemulsions	Turmeric nanoemulsion with particle size of 171.3 ± 0.52 nm, PDI of 0.25, and zeta potential of -1.23 ± 0.16 mV; biogenic silver nanoparticles with size of 141.3 ± 0.78 nm, PDI of 0.18 ± 0.03, and zeta potential of −1.23 ± 0.16 mV	**Concentrations:** 10-fold diluted turmeric nanoemulsion and biogenic silver nanoparticles at 31.3 µg/mL. **Species:** Watermelon (*Citrullus lanatus* (Thunb.) Matsum. & Nakai). **Effects:** Seed and seedlings vigor ↑, Enzymatic activity ↑, Plant morphology ↑, Biochemical activity ↑, and Harvest ↑	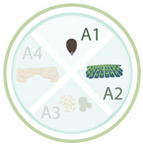	[12]
Multi-walled carbon nanotubes	Nanotubes with diameter of 13–14 nm	**Concentrations:** 70, 80, and 90 µg/mL. **Species:** Wheat (*Triticum aestivum* L.). **Effects:** Seed and seedlings vigor ↑, Plant morphology ↑, and Harvest ↑	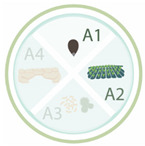	[76]
Nano-pyrite (FeS_2_)	Nanoparticles with sizes in the range 25–100 nm, with spherical morphology	**Concentrations:** 50 µg/mL. **Species:** Rice (*Oryza sativa* L.). **Effects:** Enzymatic activity ↑, Seed and seedlings vigor ↑, and Fertilizer ↓	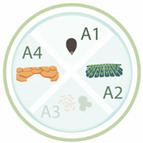	[77]
Mesoporous silica nanoparticles containing cinnamon essential oil	Nanoparticles with size of ~100 nm and pores of around 2–2.8 nm	**Concentration:** 2 mg/mL. **Species:** Pea seeds (*Pisum sativum* L.). **Effects:** Seed and seedlings vigor ↑, and antimicrobial activity ↓	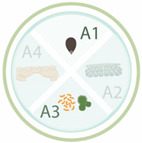	[78]
Copper and iron nanoparticles	Copper nanoparticles with sizes of around 15–30 nm; iron nanoparticles with sizes of around 20–30 nm	**Concentrations:** 20, 25, 30, 35, and 40 ppm **Species:** Wheat (*Triticum aestivum* L.) seeds of varieties galaxy-13, Pakistan-13, and NARC-11. **Effects:** Enzymatic activity ↑, Biochemical activity ↑ Anti-oxidant activity ↑, Abiotic stress resistance ↑, and Harvest ↑↓ (dose dependent)	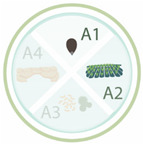	[79]
Cobalt and molybdenum oxide nanoparticles	Both types of nanoparticle had sizes of 60–80 nm and spherical morphology	**Concentrations:** 1 L of stock commercial solution for 40 kg of seeds. **Species:** Soybean seeds (*Glycine max* (L). Merr.). **Effects:** Seed and seedlings vigor ↑, Plant morphology ↑, Biomass ↑, and Enzymatic activity ↑	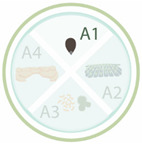	[11]
Biochar nanoparticles produced from rice and wood sawdust	Nanoparticles with sizes of ~22–56 nm and spherical morphology	**Concentrations:** 0.5, 5, and 50 mg/L. **Species:** Rice (*Oryza sativa* L.) and tomato (*Lycopersicon esculentum* Mill.). **Effects:** Seed and seedlings vigor ↑, Plant morphology ↑, and Biomass ↑	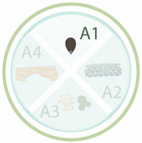	[80]
Chitosan nanoparticles loaded with thiamine	Nanoparticles with size of 560 nm, polydispersity <1, and zeta potential of 37.7 mV	**Concentration:** 0.1%. **Species:** Chickpea (*Cicer arietinum* L.) **Effects:** Seed and seedlings vigor ↑, Plant morphology ↑, and Plant growth regulator (auxins) ↑	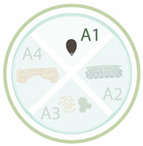	[81]
Lignin nanoparticles loaded with gibberellic acid	Nanoparticles with sizes around 200–250 nm, polydispersity of 0.17–0.38, and spherical morphology	**Concentrations:** 0.5, 1, and 1.5 mg/mL. **Species:** Arugula (*Eruca visicaria* (L.) Cav. subsp. *sativa*), tomato (*Solanum lycopersicum* L. cv. Ciliegino), and chickpea (*Cicer arietinum* L.). **Effects:** Seed and seedlings vigor ↑ (The effects varied according to the time of sowing after the treatment.)	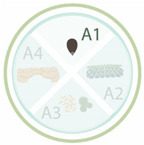	[54]
Chitosan nanoparticles loaded with gibberellic acid	Alginate/chitosan nanoparticles with 450 ± 10 nm, PDI of 0.3 and zeta potential of −29 mV. Chitosan/tripolyphosphate nanoparticles with 195 ± 1 nm, PDI of 0.3, and zeta potential of 27 mV. Spherical morphology	**Concentrations:** 0.05, 0.005, and 0.0005 mg/mL. **Species:** tomato (*Solanum lycopersicum* var. *cerasiforme*). **Effects:** Seed and seedlings vigor ↑, Plant morphology ↑, and Harvest ↑	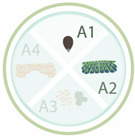	[82]
Biogenic silver nanoparticles produced using the fungus *Macrophomina phaseolina*	Nanoparticles with size of 5–30 nm	**Concentrations:** 10, 20, and 50 µg/mL. **Species:** Soybean (*Glycine* max (L.) Merr.). **Effects:** Antimicrobial activity ↑	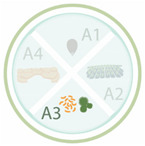	[83]
Chitosan nanoparticles	Nanoparticles with size of 400 nm and spherical morphology	**Concentration:** 250 mg/kg. **Species:** Pearl millet (*Pennisetum glaucum* (L.) R.Br.). **Effects:** Seed and seedlings vigor ↑, Antimicrobial activity ↑, Enzymatic activity ↑, Biochemical activity ↑, and Plant growth regulators ↑	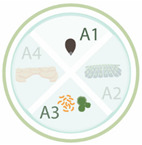	[61]
Biogenic zinc oxide nanoparticles produced using *Eclipta alba* extracts	Nanoparticles with size of 32 nm	**Concentrations:** 50, 100, 150, 200, 250, and 500 ppm. **Species:** Pearl millet (*Pennisetum glaucum* (L.) R.Br.). **Effects:** Seed and seedlings vigor ↑, Plant morphology ↑, and Antimicrobial resistance ↑	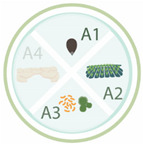	[84]
Chitosan nanoparticles containing copper	Nanoparticles with size of 374.3 ± 8.2 nm, and zeta potential of 22.6 mV	**Concentrations:** 0.01, 0.04, 0.08, 0.12, and 0.16% *w*/*v*. **Species:** Maize seeds (*Zea mays* L.). **Effects:** Seed and seedlings vigor ↑↓, Enzymatic activity ↑↓ (Dose dependent)	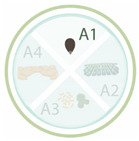	[85]
Chitosan nanoparticles containing zinc	Nanoparticles with size of 387.7 ± 4 nm, spherical morphology, polydispersity of 0.22, and zeta potential of +34 mV	**Concentrations:** 0.01, 0.04, 0.08, 0.12, and 0.16% *w*/*v*. **Species:** Maize seeds (*Zea mays* L.). **Effects:** Seed and seedlings vigor ↑, Enzymatic activity ↑, Anti-oxidant enzymes ↑, Biotic resistance ↑, and Harvest ↑	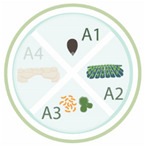	[86]
Molybdenum nanoparticles combined with the bacteria *Mesorhizobium ciceri* ST282 and *Bacillus subtilis* Ch13	Nanoparticles with size of 35–50 nm	**Concentrations:** 10 mg/L of nanoparticles, 10^8^ CFU/mL^−^ of *M. cicero* ST282, and 10^7^ CFU/mL of *B. subtilis* Ch13. **Species:** Chickpea (*Cicer arietinum* L.). **Effects:** Seed and seedlings vigor ↑, Plant morphology ↑ Anti-oxidant enzymes ↑, and Harvest ↑		[87]

## References

[1] De La Torre-Roche R., Cantu J., Tamez C., Zuverza-Mena N., Hamdi H., Adisa I.O., Elmer W., Gardea-Torresdey J., White J.C. (2020). Seed Biofortification by Engineered Nanomaterials: A Pathway To Alleviate Malnutrition?. J. Agric. Food Chem..

[2] Kah M., Tufenkji N., White J.C. (2019). Nano-Enabled Strategies to Enhance Crop Nutrition and Protection. Nat. Nanotechnol..

[3] Lowry G.V., Avellan A., Gilbertson L.M. (2019). Opportunities and Challenges for Nanotechnology in the Agri-Tech Revolution. Nat. Nanotechnol..

[4] Zhao L., Lu L., Wang A., Zhang H., Huang M., Wu H., Xing B., Wang Z., Ji R. (2020). Nano-Biotechnology in Agriculture: Use of Nanomaterials to Promote Plant Growth and Stress Tolerance. J. Agric. Food Chem..

[5] Scott N.R., Chen H., Cui H. (2018). Nanotechnology Applications and Implications of Agrochemicals toward Sustainable Agriculture and Food Systems. J. Agric. Food Chem..

[6] Fraceto L.F., Grillo R., de Medeiros G.A., Scognamiglio V., Rea G., Bartolucci C. (2016). Nanotechnology in Agriculture: Which Innovation Potential Does It Have?. Front. Environ. Sci..

[7] Panpatte D.G., Jhala Y.K., Shelat H.N., Vyas R.V., Singh D.P., Singh H.B., Prabha R. (2016). Nanoparticles: The Next Generation Technology for Sustainable Agriculture. Microbial Inoculants in Sustainable Agricultural Productivity.

[8] Camara M.C., Campos E.V.R., Monteiro R.A., Pereira A.E.S., de Freitas Proença P.L., Fraceto L.F. (2019). Development of Stimuli-Responsive Nano-Based Pesticides: Emerging Opportunities for Agriculture. J. Nanobiotechnol..

[9] Shakiba S., Astete C.E., Paudel S., Sabliov C.M., Rodrigues D.F., Louie S.M. (2020). Emerging Investigator Series: Polymeric Nanocarriers for Agricultural Applications: Synthesis, Characterization, and Environmental and Biological Interactions. Environ. Sci. Nano.

[10] Malik A., Mor V.S., Tokas J., Punia H., Malik S., Malik K., Sangwan S., Tomar S., Singh P., Singh N. (2021). Biostimulant-Treated Seedlings under Sustainable Agriculture: A Global Perspective Facing Climate Change. Agronomy.

[11] Chau N.H., Doan Q.H., Chu T.H., Nguyen T.T., Dao Trong H., Ngo Q.B. (2019). Effects of Different Nanoscale Microelement-Containing Formulations for Presowing Seed Treatment on Growth of Soybean Seedlings. J. Chem..

[12] Acharya P., Jayaprakasha G.K., Crosby K.M., Jifon J.L., Patil B.S. (2020). Nanoparticle-Mediated Seed Priming Improves Germination, Growth, Yield, and Quality of Watermelons (*Citrullus lanatus*) at Multi-Locations in Texas. Sci. Rep..

[13] Pérez-de-Luque A. (2017). Interaction of Nanomaterials with Plants: What Do We Need for Real Applications in Agriculture?. Front. Environ. Sci..

[14] Mahakham W., Sarmah A.K., Maensiri S., Theerakulpisut P. (2017). Nanopriming Technology for Enhancing Germination and Starch Metabolism of Aged Rice Seeds Using Phytosynthesized Silver Nanoparticles. Sci. Rep..

[15] Pelegrino M.T., Kohatsu M.Y., Seabra A.B., Monteiro L.R., Gomes D.G., Oliveira H.C., Rolim W.R., de Jesus T.A., Batista B.L., Lange C.N. (2020). Effects of Copper Oxide Nanoparticles on Growth of Lettuce (*Lactuca sativa* L.) Seedlings and Possible Implications of Nitric Oxide in Their Antioxidative Defense. Environ. Monit. Assess..

[16] Hayes K.L., Mui J., Song B., Sani E.S., Eisenman S.W., Sheffield J.B., Kim B. (2020). Effects, Uptake, and Translocation of Aluminum Oxide Nanoparticles in Lettuce: A Comparison Study to Phytotoxic Aluminum Ions. Sci. Total Environ..

[17] Falco W.F., Scherer M.D., Oliveira S.L., Wender H., Colbeck I., Lawson T., Caires A.R.L. (2020). Phytotoxicity of Silver Nanoparticles on Vicia Faba: Evaluation of Particle Size Effects on Photosynthetic Performance and Leaf Gas Exchange. Sci. Total Environ..

[18] Abbasi Khalaki M., Moameri M., Asgari Lajayer B., Astatkie T. (2020). Influence of Nano-Priming on Seed Germination and Plant Growth of Forage and Medicinal Plants. Plant Growth Regul..

[19] Acharya P., Jayaprakasha G.K., Crosby K.M., Jifon J.L., Patil B.S. (2019). Green-Synthesized Nanoparticles Enhanced Seedling Growth, Yield, and Quality of Onion (*Allium cepa* L.). ACS Sustain. Chem. Eng..

[20] Hu P., An J., Faulkner M.M., Wu H., Li Z., Tian X., Giraldo J.P. (2020). Nanoparticle Charge and Size Control Foliar Delivery Efficiency to Plant Cells and Organelles. ACS Nano.

[21] Bombo A.B., Pereira A.E.S., Lusa M.G., de Medeiros Oliveira E., de Oliveira J.L., Campos E.V.R., de Jesus M.B., Oliveira H.C., Fraceto L.F., Mayer J.L.S. (2019). A Mechanistic View of Interactions of a Nanoherbicide with Target Organism. J. Agric. Food Chem..

[22] Palocci C., Valletta A., Chronopoulou L., Donati L., Bramosanti M., Brasili E., Baldan B., Pasqua G. (2017). Endocytic Pathways Involved in PLGA Nanoparticle Uptake by Grapevine Cells and Role of Cell Wall and Membrane in Size Selection. Plant Cell Rep..

[23] Valletta A., Chronopoulou L., Palocci C., Baldan B., Donati L., Pasqua G. (2014). Poly(Lactic-Co-Glycolic) Acid Nanoparticles Uptake by Vitis Vinifera and Grapevine-Pathogenic Fungi. J. Nanoparticle Res..

[24] Spielman-Sun E., Avellan A., Bland G.D., Tappero R.V., Acerbo A.S., Unrine J.M., Giraldo J.P., Lowry G.V. (2019). Nanoparticle Surface Charge Influences Translocation and Leaf Distribution in Vascular Plants with Contrasting Anatomy. Environ. Sci. Nano.

[25] Avellan A., Schwab F., Masion A., Chaurand P., Borschneck D., Vidal V., Rose J., Santaella C., Levard C. (2017). Nanoparticle Uptake in Plants: Gold Nanomaterial Localized in Roots of Arabidopsis Thaliana by X-Ray Computed Nanotomography and Hyperspectral Imaging. Environ. Sci. Technol..

[26] Avellan A., Yun J., Zhang Y., Spielman-Sun E., Unrine J.M., Thieme J., Li J., Lombi E., Bland G., Lowry G.V. (2019). Nanoparticle Size and Coating Chemistry Control Foliar Uptake Pathways, Translocation, and Leaf-to-Rhizosphere Transport in Wheat. ACS Nano.

[27] An J., Hu P., Li F., Wu H., Shen Y., White J.C., Tian X., Li Z., Giraldo J.P. (2020). Emerging Investigator Series: Molecular Mechanisms of Plant Salinity Stress Tolerance Improvement by Seed Priming with Cerium Oxide Nanoparticles. Environ. Sci. Nano.

[28] Salama D.M. (2019). Effect of Zinc Oxide Nanoparticles on the Growth, Genomic DNA, Production and the Quality of Common Dry Bean (*Phaseolus vulgaris*). Biocatal. Agric. Biotechnol..

[29] Ye Y., Cota-Ruiz K., Hernández-Viezcas J.A., Valdés C., Medina-Velo I.A., Turley R.S., Peralta-Videa J.R., Gardea-Torresdey J.L. (2020). Manganese Nanoparticles Control Salinity-Modulated Molecular Responses in *Capsicum annuum* L. through Priming: A Sustainable Approach for Agriculture. ACS Sustain. Chem. Eng..

[30] Hussain A., Rizwan M., Ali Q., Ali S. (2019). Seed Priming with Silicon Nanoparticles Improved the Biomass and Yield While Reduced the Oxidative Stress and Cadmium Concentration in Wheat Grains. Environ. Sci. Pollut. Res..

[31] Kasote D.M., Lee J.H.J., Jayaprakasha G.K., Patil B.S. (2019). Seed Priming with Iron Oxide Nanoparticles Modulate Antioxidant Potential and Defense-Linked Hormones in Watermelon Seedlings. ACS Sustain. Chem. Eng..

[32] Pirzada T., de Farias B.V., Mathew R., Guenther R.H., Byrd M.V., Sit T.L., Pal L., Opperman C.H., Khan S.A. (2020). Recent Advances in Biodegradable Matrices for Active Ingredient Release in Crop Protection: Towards Attaining Sustainability in Agriculture. Curr. Opin. Colloid Interface Sci..

[33] Savassa S.M., Duran N.M., Rodrigues E.S., de Almeida E., van Gestel C.A.M., Bompadre T.F.V., de Carvalho H.W.P. (2018). Effects of ZnO Nanoparticles on Phaseolus Vulgaris Germination and Seedling Development Determined by X-Ray Spectroscopy. ACS Appl. Nano Mater..

[34] Mahakham W., Theerakulpisut P., Maensiri S., Phumying S., Sarmah A.K. (2016). Environmentally Benign Synthesis of Phytochemicals-Capped Gold Nanoparticles as Nanopriming Agent for Promoting Maize Seed Germination. Sci. Total Environ..

[35] Chandrasekaran U., Luo X., Wang Q., Shu K. (2020). Are There Unidentified Factors Involved in the Germination of Nanoprimed Seeds?. Front. Plant Sci..

[36] Nonogaki H., Bassel G.W., Bewley J.D. (2010). Germination—Still a Mystery. Plant Sci..

[37] Nonogaki H. (2014). Seed Dormancy and Germination—Emerging Mechanisms and New Hypotheses. Front. Plant Sci..

[38] Ibrahim E.A. (2016). Seed Priming to Alleviate Salinity Stress in Germinating Seeds. J. Plant Physiol..

[39] Oracz K., Karpiński S. (2016). Phytohormones Signaling Pathways and ROS Involvement in Seed Germination. Front. Plant Sci..

[40] Choudhary A., Kumar A., Kaur N. (2020). ROS and Oxidative Burst: Roots in Plant Development. Plant Divers..

[41] Bailly C. (2019). The Signalling Role of ROS in the Regulation of Seed Germination and Dormancy. Biochem. J..

[42] Wu M., Wu J., Gan Y. (2020). The New Insight of Auxin Functions: Transition from Seed Dormancy to Germination and Floral Opening in Plants. Plant Growth Regul..

[43] Shuai H., Meng Y., Luo X., Chen F., Qi Y., Yang W., Shu K. (2016). The Roles of Auxin in Seed Dormancy and Germination. Yi Chuan Hered..

[44] Bourioug M., Ezzaza K., Bouabid R., Alaoui-Mhamdi M., Bungau S., Bourgeade P., Alaoui-Sossé L., Alaoui-Sossé B., Aleya L. (2020). Influence of Hydro- and Osmo-Priming on Sunflower Seeds to Break Dormancy and Improve Crop Performance under Water Stress. Environ. Sci. Pollut. Res..

[45] Jisha K.C., Vijayakumari K., Puthur J.T. (2013). Seed Priming for Abiotic Stress Tolerance: An Overview. Acta Physiol. Plant..

[46] Thomas T.T.D., Puthur J.T. (2017). UV Radiation Priming: A Means of Amplifying the Inherent Potential for Abiotic Stress Tolerance in Crop Plants. Environ. Exp. Bot..

[47] Carrillo-Reche J., Vallejo-Marín M., Quilliam R.S. (2018). Quantifying the Potential of ‘on-Farm’ Seed Priming to Increase Crop Performance in Developing Countries. A Meta-Analysis. Agron. Sustain. Dev..

[48] Lemmens E., Deleu L.J., De Brier N., De Man W.L., De Proft M., Prinsen E., Delcour J.A. (2019). The Impact of Hydro-Priming and Osmo-Priming on Seedling Characteristics, Plant Hormone Concentrations, Activity of Selected Hydrolytic Enzymes, and Cell Wall and Phytate Hydrolysis in Sprouted Wheat (*Triticum aestivum* L.). ACS Omega.

[49] Noorhosseini S.A., Jokar N.K., Damalas C.A. (2018). Improving Seed Germination and Early Growth of Garden Cress (*Lepidium sativum*) and Basil (*Ocimum basilicum*) with Hydro-Priming. J. Plant Growth Regul..

[50] Saddiq M.S., Iqbal S., Afzal I., Ibrahim A.M.H., Bakhtavar M.A., Jahanzaib M.B.H., Maqbool M.M. (2019). Mitigation of Salinity Stress in Wheat (*Triticum aestivum* L.) Seedlings through Physiological Seed Enhancements. J. Plant Nutr..

[51] Sytar O., Kumari P., Yadav S., Brestic M., Rastogi A. (2019). Phytohormone Priming: Regulator for Heavy Metal Stress in Plants. J. Plant Growth Regul..

[52] Shukla P., Chaurasia P., Younis K., Qadri O.S., Faridi S.A., Srivastava G. (2019). Nanotechnology in Sustainable Agriculture: Studies from Seed Priming to Post-Harvest Management. Nanotechnol. Environ. Eng..

[53] Duran N.M., Savassa S.M., Lima R.G.D., de Almeida E., Linhares F.S., van Gestel C.A.M., Pereira de Carvalho H.W. (2017). X-Ray Spectroscopy Uncovering the Effects of Cu Based Nanoparticle Concentration and Structure on *Phaseolus vulgaris* Germination and Seedling Development. J. Agric. Food Chem..

[54] Falsini S., Clemente I., Papini A., Tani C., Schiff S., Salvatici M.C., Petruccelli R., Benelli C., Giordano C., Gonnelli C. (2019). When Sustainable Nanochemistry Meets Agriculture: Lignin Nanocapsules for Bioactive Compound Delivery to Plantlets. ACS Sustain. Chem. Eng..

[55] Montanha G.S., Rodrigues E.S., Marques J.P.R., de Almeida E., Colzato M., Pereira de Carvalho H.W. (2020). Zinc Nanocoated Seeds: An Alternative to Boost Soybean Seed Germination and Seedling Development. SN Appl. Sci..

[56] Gross M.S., Bean T.G., Hladik M.L., Rattner B.A., Kuivila K.M. (2020). Uptake, Metabolism, and Elimination of Fungicides from Coated Wheat Seeds in Japanese Quail (*Coturnix japonica*). J. Agric. Food Chem..

[57] Khodakovskaya M., Dervishi E., Mahmood M., Xu Y., Li Z., Watanabe F., Biris A.S. (2009). Carbon Nanotubes Are Able To Penetrate Plant Seed Coat and Dramatically Affect Seed Germination and Plant Growth. ACS Nano.

[58] Khodakovskaya M.V., Kim B.-S., Kim J.N., Alimohammadi M., Dervishi E., Mustafa T., Cernigla C.E. (2013). Carbon Nanotubes as Plant Growth Regulators: Effects on Tomato Growth, Reproductive System, and Soil Microbial Community. Small.

[59] Lahiani M.H., Dervishi E., Chen J., Nima Z., Gaume A., Biris A.S., Khodakovskaya M.V. (2013). Impact of Carbon Nanotube Exposure to Seeds of Valuable Crops. ACS Appl. Mater. Interfaces.

[60] Villagarcia H., Dervishi E., de Silva K., Biris A.S., Khodakovskaya M.V. (2012). Surface Chemistry of Carbon Nanotubes Impacts the Growth and Expression of Water Channel Protein in Tomato Plants. Small.

[61] Siddaiah C.N., Prasanth K.V.H., Satyanarayana N.R., Mudili V., Gupta V.K., Kalagatur N.K., Satyavati T., Dai X.-F., Chen J.-Y., Mocan A. (2018). Chitosan Nanoparticles Having Higher Degree of Acetylation Induce Resistance against Pearl Millet Downy Mildew through Nitric Oxide Generation. Sci. Rep..

[62] Li R., He J., Xie H., Wang W., Bose S.K., Sun Y., Hu J., Yin H. (2019). Effects of Chitosan Nanoparticles on Seed Germination and Seedling Growth of Wheat (*Triticum aestivum* L.). Int. J. Biol. Macromol..

[63] Abdel Latef A.A.H., Abu Alhmad M.F., Abdelfattah K.E. (2017). The Possible Roles of Priming with ZnO Nanoparticles in Mitigation of Salinity Stress in Lupine (*Lupinus termis*) Plants. J. Plant Growth Regul..

[64] Itroutwar P.D., Govindaraju K., Tamilselvan S., Kannan M., Raja K., Subramanian K.S. (2019). Seaweed-Based Biogenic ZnO Nanoparticles for Improving Agro-Morphological Characteristics of Rice (*Oryza sativa* L.). J. Plant Growth Regul..

[65] Maswada H.F., Djanaguiraman M., Prasad P.V.V. (2018). Seed Treatment with Nano-Iron (III) Oxide Enhances Germination, Seeding Growth and Salinity Tolerance of Sorghum. J. Agron. Crop Sci..

[66] Rizwan M., Ali S., Ali B., Adrees M., Arshad M., Hussain A., Zia ur Rehman M., Waris A.A. (2019). Zinc and Iron Oxide Nanoparticles Improved the Plant Growth and Reduced the Oxidative Stress and Cadmium Concentration in Wheat. Chemosphere.

[67] Dileep Kumar G., Raja K., Natarajan N., Govindaraju K., Subramanian K.S. (2020). Invigouration Treatment of Metal and Metal Oxide Nanoparticles for Improving the Seed Quality of Aged Chilli Seeds (*Capsicum annum* L.). Mater. Chem. Phys..

[68] Ahuja R., Sidhu A., Bala A. (2019). Synthesis and Evaluation of Iron (Ii) Sulfide Aqua Nanoparticles (FeS-NPs) against Fusarium Verticillioides Causing Sheath Rot and Seed Discoloration of Rice. Eur. J. Plant Pathol..

[69] Abdel-Aziz H.M.M., Hasaneen M.N.A., Omer A.M. (2019). Impact of Engineered Nanomaterials Either Alone or Loaded with NPK on Growth and Productivity of French Bean Plants: Seed Priming vs Foliar Application. S. Afr. J. Bot..

[70] Divya K., Vijayan S., Nair S.J., Jisha M.S. (2019). Optimization of Chitosan Nanoparticle Synthesis and Its Potential Application as Germination Elicitor of *Oryza sativa* L.. Int. J. Biol. Macromol..

[71] Rahman M.S., Chakraborty A., Mazumdar S., Nandi N.C., Bhuiyan M.N.I., Alauddin S.M., Khan I.A., Hossain M.J. (2020). Effects of Poly (Vinylpyrrolidone) Protected Platinum Nanoparticles on Seed Germination and Growth Performance of Pisum Sativum. Nano Struct. Nano Objects.

[72] Kannaujia R., Srivastava C.M., Prasad V., Singh B.N., Pandey V. (2019). Phyllanthus Emblica Fruit Extract Stabilized Biogenic Silver Nanoparticles as a Growth Promoter of Wheat Varieties by Reducing ROS Toxicity. Plant Physiol. Biochem..

[73] Sathiyabama M., Muthukumar S. (2020). Chitosan Guar Nanoparticle Preparation and Its in Vitro Antimicrobial Activity towards Phytopathogens of Rice. Int. J. Biol. Macromol..

[74] Sundaria N., Singh M., Upreti P., Chauhan R.P., Jaiswal J.P., Kumar A. (2019). Seed Priming with Iron Oxide Nanoparticles Triggers Iron Acquisition and Biofortification in Wheat (*Triticum aestivum* L.) Grains. J. Plant Growth Regul..

[75] Guha T., Ravikumar K.V.G., Mukherjee A., Mukherjee A., Kundu R. (2018). Nanopriming with Zero Valent Iron (NZVI) Enhances Germination and Growth in Aromatic Rice Cultivar (*Oryza sativa* Cv. Gobindabhog L.). Plant Physiol. Biochem..

[76] Joshi A., Kaur S., Dharamvir K., Nayyar H., Verma G. (2018). Multi-Walled Carbon Nanotubes Applied through Seed-Priming Influence Early Germination, Root Hair, Growth and Yield of Bread Wheat (*Triticum aestivum* L.): Multiwalled Carbon Nanotube Influence on Bread Wheat. J. Sci. Food Agric..

[77] Das C.K., Jangir H., Kumar J., Verma S., Mahapatra S.S., Philip D., Srivastava G., Das M. (2018). Nano-Pyrite Seed Dressing: A Sustainable Design for NPK Equivalent Rice Production. Nanotechnol. Environ. Eng..

[78] Bravo Cadena M., Preston G.M., Van der Hoorn R.A.L., Flanagan N.A., Townley H.E., Thompson I.P. (2018). Enhancing Cinnamon Essential Oil Activity by Nanoparticle Encapsulation to Control Seed Pathogens. Ind. Crops Prod..

[79] Yasmeen F., Raja N.I., Razzaq A., Komatsu S. (2017). Proteomic and Physiological Analyses of Wheat Seeds Exposed to Copper and Iron Nanoparticles. Biochim. Biophys. Acta BBA Proteins Proteom..

[80] Zhang K., Wang Y., Mao J., Chen B. (2020). Effects of Biochar Nanoparticles on Seed Germination and Seedling Growth. Environ. Pollut..

[81] Muthukrishnan S., Murugan I., Selvaraj M. (2019). Chitosan Nanoparticles Loaded with Thiamine Stimulate Growth and Enhances Protection against Wilt Disease in Chickpea. Carbohydr. Polym..

[82] Pereira A.E.S., Oliveira H.C., Fraceto L.F. (2019). Polymeric Nanoparticles as an Alternative for Application of Gibberellic Acid in Sustainable Agriculture: A Field Study. Sci. Rep..

[83] Spagnoletti F.N., Spedalieri C., Kronberg F., Giacometti R. (2019). Extracellular Biosynthesis of Bactericidal Ag/AgCl Nanoparticles for Crop Protection Using the Fungus Macrophomina Phaseolina. J. Environ. Manag..

[84] Nandhini M., Rajini S.B., Udayashankar A.C., Niranjana S.R., Lund O.S., Shetty H.S., Prakash H.S. (2019). Biofabricated Zinc Oxide Nanoparticles as an Eco-Friendly Alternative for Growth Promotion and Management of Downy Mildew of Pearl Millet. Crop Prot..

[85] Saharan V., Kumaraswamy R.V., Choudhary R.C., Kumari S., Pal A., Raliya R., Biswas P. (2016). Cu-Chitosan Nanoparticle Mediated Sustainable Approach To Enhance Seedling Growth in Maize by Mobilizing Reserved Food. J. Agric. Food Chem..

[86] Choudhary R.C., Kumaraswamy R.V., Kumari S., Sharma S.S., Pal A., Raliya R., Biswas P., Saharan V. (2019). Zinc Encapsulated Chitosan Nanoparticle to Promote Maize Crop Yield. Int. J. Biol. Macromol..

[87] Shcherbakova E.N., Shcherbakov A.V., Andronov E.E., Gonchar L.N., Kalenskaya S.M., Chebotar V.K. (2017). Combined Pre-Seed Treatment with Microbial Inoculants and Mo Nanoparticles Changes Composition of Root Exudates and Rhizosphere Microbiome Structure of Chickpea (*Cicer arietinum* L.) Plants. Symbiosis.

[88] Kumar S., Nehra M., Dilbaghi N., Marrazza G., Tuteja S.K., Kim K.-H. (2020). Nanovehicles for Plant Modifications towards Pest- and Disease-Resistance Traits. Trends Plant Sci..

[89] De Oliveira J.L., Campos E.V.R., Germano-Costa T., Lima R., Vechia J.F.D., Soares S.T., de Andrade D.J., Gonçalves K.C., do Nascimento J., Polanczyk R.A. (2019). Association of Zein Nanoparticles with Botanical Compounds for Effective Pest Control Systems. Pest Manag. Sci..

[90] Raja K., Sowmya R., Sudhagar R., Moorthy P.S., Govindaraju K., Subramanian K.S. (2019). Biogenic ZnO and Cu Nanoparticles to Improve Seed Germination Quality in Blackgram (*Vigna mungo*). Mater. Lett..

[91] Maroufpour N., Mousavi M., Abbasi M., Ghorbanpour M., Ghorbanpour M., Bhargava P., Varma A., Choudhary D.K. (2020). Biogenic Nanoparticles as Novel Sustainable Approach for Plant Protection. Biogenic Nano-Particles and Their Use in Agro-Ecosystems.

[92] Fraceto L.F., Maruyama C.R., Guilger M., Mishra S., Keswani C., Singh H.B., Lima R.D. (2018). Trichoderma Harzianum-Based Novel Formulations: Potential Applications for Management of Next-Gen Agricultural Challenges. J. Chem. Technol. Biotechnol..

[93] Gao Y., Liang Y., Dong H., Niu J., Tang J., Yang J., Tang G., Zhou Z., Tang R., Shi X. (2020). A Bioresponsive System Based on Mesoporous Organosilica Nanoparticles for Smart Delivery of Fungicide in Response to Pathogen Presence. ACS Sustain. Chem. Eng..

[94] Maluin F.N., Hussein M.Z., Yusof N.A., Fakurazi S., Idris A.S., Hilmi N.H.Z., Daim L.D.J. (2020). Phytotoxicity of Chitosan-Based Agronanofungicides in the Vegetative Growth of Oil Palm Seedling. PLoS ONE.

[95] Kavetsou E., Koutsoukos S., Daferera D., Polissiou M.G., Karagiannis D., Perdikis D.C., Detsi A. (2019). Encapsulation of *Mentha pulegium* Essential Oil in Yeast Cell Microcarriers: An Approach to Environmentally Friendly Pesticides. J. Agric. Food Chem..

[96] Campos E.V.R., Proença P.L.F., Oliveira J.L., Bakshi M., Abhilash P.C., Fraceto L.F. (2019). Use of Botanical Insecticides for Sustainable Agriculture: Future Perspectives. Ecol. Indic..

[97] Pereira A.E.S., Silva P.M., Oliveira J.L., Oliveira H.C., Fraceto L.F. (2017). Chitosan Nanoparticles as Carrier Systems for the Plant Growth Hormone Gibberellic Acid. Colloids Surf. B Biointerfaces.

[98] Yi Z., Hussain H.I., Feng C., Sun D., She F., Rookes J.E., Cahill D.M., Kong L. (2015). Functionalized Mesoporous Silica Nanoparticles with Redox-Responsive Short-Chain Gatekeepers for Agrochemical Delivery. ACS Appl. Mater. Interfaces.

[99] Guo H., White J.C., Wang Z., Xing B. (2018). Nano-Enabled Fertilizers to Control the Release and Use Efficiency of Nutrients. Curr. Opin. Environ. Sci. Health.

[100] Du B.D., Ngoc D.T.B., Thang N.D., Tuan L.N.A., Thach B.D., Hien N.Q. (2019). Synthesis and in Vitro Antifungal Efficiency of Alginate-Stabilized Cu_2_O-Cu Nanoparticles against *Neoscytalidium dimidiatum* Causing Brown Spot Disease on Dragon Fruit Plants (*Hylocereus undatus*). Vietnam J. Chem..

[101] Ji M., Sun X., Guo X., Zhu W., Wu J., Chen L., Wang J., Chen M., Cheng C., Zhang Q. (2019). Green Synthesis, Characterization and in Vitro Release of Cinnamaldehyde/Sodium Alginate/Chitosan Nanoparticles. Food Hydrocoll..

[102] Qi T., Lü S., Zhang S.-F., Bai X., Chen J., Huang M., Liu M. (2020). Zein Coated Porous Carboxymethyl Starch Fertilizer for Iron Promoting and Phosphate Sustainable Release. J. Clean. Prod..

[103] Coelho C.C.S., Michelin M., Cerqueira M.A., Gonçalves C., Tonon R.V., Pastrana L.M., Freitas-Silva O., Vicente A.A., Cabral L.M.C., Teixeira J.A. (2018). Cellulose Nanocrystals from Grape Pomace: Production, Properties and Cytotoxicity Assessment. Carbohydr. Polym..

[104] Zhang H., Yang M., Luan Q., Tang H., Huang F., Xiang X., Yang C. (2017). Cellulose Anionic Hydrogels Based on Cellulose Nanofibers as Natural Stimulants for Seed Germination and Seedling Growth. J. Agric. Food Chem..

[105] Campos E.V.R., Oliveira J.L.D., da Silva C.M.G., Pascoli M., Pasquoto T., Lima R., Abhilash P.C., Fernandes Fraceto L. (2015). Polymeric and Solid Lipid Nanoparticles for Sustained Release of Carbendazim and Tebuconazole in Agricultural Applications. Sci. Rep..

[106] Da Silva Santos V., Badan Ribeiro A.P., Andrade Santana M.H. (2019). Solid Lipid Nanoparticles as Carriers for Lipophilic Compounds for Applications in Foods. Food Res. Int..

[107] Kashyap P.L., Xiang X., Heiden P. (2015). Chitosan Nanoparticle Based Delivery Systems for Sustainable Agriculture. Int. J. Biol. Macromol..

[108] Chandra S., Chakraborty N., Dasgupta A., Sarkar J., Panda K., Acharya K. (2015). Chitosan Nanoparticles: A Positive Modulator of Innate Immune Responses in Plants. Sci. Rep..

[109] Jiménez-Arias D., Morales-Sierra S., Borges A.A., Díaz Díaz D. (2020). Biostimulant Nanoencapsulation: The New Keystone To Fight Hunger. J. Agric. Food Chem..

[110] Lombardo D., Kiselev M.A., Caccamo M.T. Smart Nanoparticles for Drug Delivery Application: Development of Versatile Nanocarrier Platforms in Biotechnology and Nanomedicine. https://www.hindawi.com/journals/jnm/2019/3702518/.

[111] Fischer J., Beckers S.J., Yiamsawas D., Thines E., Landfester K., Wurm F.R. (2019). Targeted Drug Delivery in Plants: Enzyme-Responsive Lignin Nanocarriers for the Curative Treatment of the Worldwide Grapevine Trunk Disease Esca. Adv. Sci..

[112] Panyuta O., Belava V., Fomaidi S., Kalinichenko O., Volkogon M., Taran N. (2016). The Effect of Pre-Sowing Seed Treatment with Metal Nanoparticles on the Formation of the Defensive Reaction of Wheat Seedlings Infected with the Eyespot Causal Agent. Nanoscale Res. Lett..

[113] Schmitt C.C., Moreira R., Neves R.C., Richter D., Funke A., Raffelt K., Grunwaldt J.-D., Dahmen N. (2020). From Agriculture Residue to Upgraded Product: The Thermochemical Conversion of Sugarcane Bagasse for Fuel and Chemical Products. Fuel Process. Technol..

[114] Matyjaszczyk E. (2017). Comparison between Seed and Foliar Treatment as a Tool in Integrated Pest Management. J. Agric. Food Chem..

[115] Farias B.V., Pirzada T., Mathew R., Sit T.L., Opperman C., Khan S.A. (2019). Electrospun Polymer Nanofibers as Seed Coatings for Crop Protection. ACS Sustain. Chem. Eng..

[116] Lowry G.V., Gregory K.B., Apte S.C., Lead J.R. (2012). Transformations of Nanomaterials in the Environment. Environ. Sci. Technol..

[117] Nguyen D.V., Nguyen H.M., Le N.T., Nguyen K.H., Le H.M., Nguyen A.T., Dinh N.T.T., Hoang S.A., Ha C.V. (2020). Copper Nanoparticle Application Enhances Plant Growth and Grain Yield in Maize under Drought Stress Conditions. Plant Biol..

[118] Barbieri M., Sappa G., Nigro A. (2018). Soil Pollution: Anthropogenic versus Geogenic Contributions over Large Areas of the Lazio Region. J. Geochem. Explor..

[119] Qayyum M.F., Rehman M.Z.U., Ali S., Rizwan M., Naeem A., Maqsood M.A., Khalid H., Rinklebe J., Ok Y.S. (2017). Residual Effects of Monoammonium Phosphate, Gypsum and Elemental Sulfur on Cadmium Phytoavailability and Translocation from Soil to Wheat in an Effluent Irrigated Field. Chemosphere.

[120] Plaksenkova I., Kokina I., Petrova A., Jermaļonoka M., Gerbreders V., Krasovska M. The Impact of Zinc Oxide Nanoparticles on Cytotoxicity, Genotoxicity, and MiRNA Expression in Barley (*Hordeum vulgare* L.) Seedlings. https://www.hindawi.com/journals/tswj/2020/6649746/.

[121] Raliya R., Tarafdar J.C., Biswas P. (2016). Enhancing the Mobilization of Native Phosphorus in the Mung Bean Rhizosphere Using ZnO Nanoparticles Synthesized by Soil Fungi. J. Agric. Food Chem..

[122] Dai Y., Chen F., Yue L., Li T., Jiang Z., Xu Z., Wang Z., Xing B. (2020). Uptake, Transport, and Transformation of CeO2 Nanoparticles by Strawberry and Their Impact on the Rhizosphere Bacterial Community. ACS Sustain. Chem. Eng..

[123] Zhang H., Huang M., Zhang W., Gardea-Torresdey J.L., White J.C., Ji R., Zhao L. (2020). Silver Nanoparticles Alter Soil Microbial Community Compositions and Metabolite Profiles in Unplanted and Cucumber-Planted Soils. Environ. Sci. Technol..

[124] Li M., Liu H., Dang F., Hintelmann H., Yin B., Zhou D. (2020). Alteration of Crop Yield and Quality of Three Vegetables upon Exposure to Silver Nanoparticles in Sludge-Amended Soil. ACS Sustain. Chem. Eng..

[125] Zhai Y., Hunting E.R., Liu G., Baas E., Peijnenburg W.J.G.M., Vijver M.G. (2019). Compositional Alterations in Soil Bacterial Communities Exposed to TiO2 Nanoparticles Are Not Reflected in Functional Impacts. Environ. Res..

[126] Chavan S., Nadanathangam V. (2020). Shifts in Metabolic Patterns of Soil Bacterial Communities on Exposure to Metal Engineered Nanomaterials. Ecotoxicol. Environ. Saf..

[127] Santaella C., Plancot B., Fraceto L.F., S.S. de Castro V.L., Grillo R., Ávila D., Caixeta Oliveira H., Lima R. (2020). Interactions of Nanoenabled Agrochemicals with Soil Microbiome. Nanopesticides: From Research and Development to Mechanisms of Action and Sustainable Use in Agriculture.

[128] Hamidat M., Barakat M., Ortet P., Chanéac C., Rose J., Bottero J.-Y., Heulin T., Achouak W., Santaella C. Design Defines the Effects of Nanoceria at a Low Dose on Soil Microbiota and the Potentiation of Impacts by the Canola Plant. https://pubs.acs.org/doi/pdf/10.1021/acs.est.6b01056.

[129] Simonin M., Colman B.P., Tang W., Judy J.D., Anderson S.M., Bergemann C.M., Rocca J.D., Unrine J.M., Cassar N., Bernhardt E.S. (2018). Plant and Microbial Responses to Repeated Cu (OH)2 Nanopesticide Exposures under Different Fertilization Levels in an Agro-Ecosystem. Front. Microbiol..

[130] Kah M., Kookana R.S., Gogos A., Bucheli T.D. (2018). A Critical Evaluation of Nanopesticides and Nanofertilizers against Their Conventional Analogues. Nat. Nanotechnol..

